# Mitochondrial transfer: a novel paradigm for wound healing

**DOI:** 10.1093/burnst/tkag043

**Published:** 2026-06-26

**Authors:** Yin He, Hong-Chao Huang, Lei Li, Si-Ya Wang, Guang-Yi Wang, Shi-Chu Xiao, Yong-Jun Zheng

**Affiliations:** Department of Burn Surgery, The First Affiliated Hospital of Naval Medical University, 168th Changhai Road,Yangpu District, Shanghai 200433, People's Republic of China; Department of Burn Surgery, The First Affiliated Hospital of Naval Medical University, 168th Changhai Road,Yangpu District, Shanghai 200433, People's Republic of China; Department of Burn Surgery, The First Affiliated Hospital of Naval Medical University, 168th Changhai Road,Yangpu District, Shanghai 200433, People's Republic of China; Department of Burn Surgery, The First Affiliated Hospital of Naval Medical University, 168th Changhai Road,Yangpu District, Shanghai 200433, People's Republic of China; Department of Burn Surgery, The First Affiliated Hospital of Naval Medical University, 168th Changhai Road,Yangpu District, Shanghai 200433, People's Republic of China; Department of Burn Surgery, The First Affiliated Hospital of Naval Medical University, 168th Changhai Road,Yangpu District, Shanghai 200433, People's Republic of China; Department of Burn Surgery, The First Affiliated Hospital of Naval Medical University, 168th Changhai Road,Yangpu District, Shanghai 200433, People's Republic of China

**Keywords:** Mitochondrial transfer, Mitochondrial transplantation, Wound healing, Mitochondria

## Abstract

Mitochondria not only serve as cellular powerhouses and metabolic regulatory hubs but also play crucial roles in calcium homeostasis, apoptosis regulation, and signal transduction. During wound repair, mitochondria modulate inflammatory and immune responses; drive angiogenesis; and supply energy for the proliferation, differentiation, and migration of repair cells such as fibroblasts and epithelial cells. When mitochondrial dysfunction exceeds the self-regulatory capacity, cellular energy deficits, metabolic disturbances, dysregulated signaling, and oxidative stress damage occur. These abnormalities collectively impair cellular repair mechanisms, ultimately contributing to chronic nonhealing wounds. Historically, mitochondria were thought to be acquired *via* vertical inheritance through cell division or mitochondrial biogenesis. However, the discovery of intercellular mitochondrial transfer provides novel insights into mitochondrial acquisition and presents new avenues for rescuing mitochondrial dysfunction. This article comprehensively reviews the dialectical relationship between mitochondrial dysfunction and impaired wound healing by integrating mechanisms of mitochondrial transfer, including modes, triggers, and regulatory pathways. It further highlights the therapeutic potential of mitochondrial transfer in wound repair. Additionally, this article provides forward-looking perspectives on clinical applications and future developments in mitochondrial transfer for wound healing, aiming to establish a theoretical foundation for mitochondrial transplantation therapies.

## Graphical Abstract

**Graphical Abstract:**
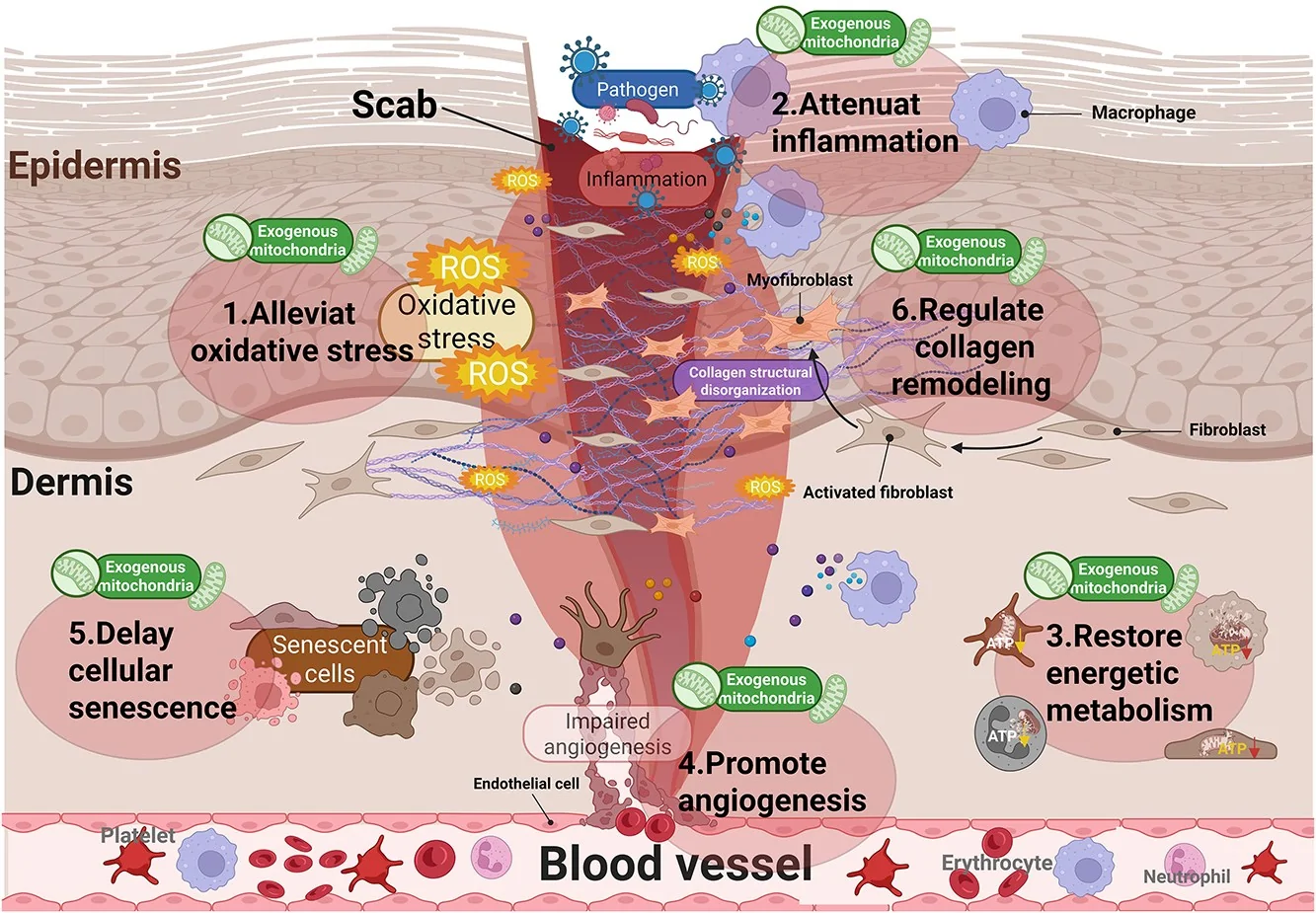

## Highlights

- Intercellular mitochondrial transfer relies on multiple biological pathways, and its initiation and efficiency are modulated by diverse trigger factors and regulatory molecules.
- Mitochondrial transfer facilitates wound repair *via* multiple mechanisms, including alleviating oxidative stress, attenuating inflammation, restoring energy metabolism, delaying cellular senescence, promoting angiogenesis, and regulating collagen remodeling.
- Derived from mitochondrial transfer, diverse mitochondrial transplantation strategies offer new options for wound therapy; yet, several obstacles remain before clinical translation.

## Background

Wound healing is a complex and orderly pathophysiological process initiated by the body in response to injury and involves the precise coordination of multiple stages, including the inflammatory response, cell proliferation, and tissue remodeling [1]. Mitochondria play a critical regulatory role throughout the entire wound healing cascade, and mitochondrial dysfunction disrupts normal healing through multiple mechanisms. During the inflammatory phase, mitochondria maintain wound inflammatory homeostasis by regulating the metabolic reprogramming of immune cells, such as macrophages. Specifically, the polarization state of macrophages is directly controlled by mitochondrial-mediated metabolic reprogramming. M1 macrophages rely on aerobic glycolysis, with suppressed mitochondrial oxidative phosphorylation (OXPHOS) and a disrupted tricarboxylic acid (TCA) cycle involving succinate and citrate, leading to the accumulation of mitochondrial reactive oxygen species (ROS) and proinflammatory metabolites. Conversely, M2 macrophages depend on intact OXPHOS and fatty acid oxidation (FAO), generating ATP and synthesizing anti-inflammatory mediators *via* the mitochondria [2]. Impaired mitochondrial function results in oxidative metabolic deficits in macrophages, an insufficient energy supply, and an inability to effectively perform FAO, thereby promoting M1 polarization and hindering M2 conversion. This imbalance in polarization leads to excessive ROS generation [3], and this excessive oxidative stress subsequently further damages mitochondrial structure and function, establishing a vicious cycle [4–7]. Furthermore, mitochondrial metabolic profiles dictate immune cell differentiation fates, proliferative capacities, and effector functions. Numerous immune cells undergo specific metabolic reprogramming during their mature differentiation stages. For instance, upon activation and differentiation from a quiescent state to effector cells, CD4^+^ T cells switch their energy metabolism from OXPHOS dominance to a mode co-fueled by aerobic glycolysis, the pentose phosphate pathway, and glutaminolysis [8, 9]. B cells can adapt to distinct cellular states, such as quiescence, proliferation, and antibody synthesis, by dynamically switching their mitochondrial metabolic profiles [10]. Besides interfering with the phenotypic conversion of immune cells, damaged mitochondrial also releases mitochondrial deoxyribonucleic acid (mtDNA), which can be recognized by intracellular pattern recognition receptors, activating nucleic acid sensing pathways such as cGAS–STING signaling. This process promotes NLRP3 inflammasome activation and the release of proinflammatory factors such as interleukin-1β (IL-1β), amplifying inflammation and trapping the wound in a state of persistent inflammation that prevents the transition to the proliferative phase [11]. In the proliferative phase, mitochondria produce adenosine triphosphate (ATP) *via* OXPHOS to fuel the proliferation and migration of key repair cells such as fibroblasts, keratinocytes, and endothelial cells (ECs) [12]. Mitochondrial dysfunction compromises this energy supply, forcing cells to switch to inefficient glycolytic metabolism, resulting in an ATP deficiency and insufficient production of biosynthetic precursors. This process inhibits granulation tissue formation, re-epithelialization, and angiogenesis, thereby delaying wound closure [13, 14]. During the remodeling phase, mitochondria regulate final tissue optimization by modulating apoptosis, fibroblast differentiation, and cellular function. When dysfunctional, mitochondria fail to properly regulate normal apoptosis, leading to delayed cell death and the persistence of granulation tissue [15]. Mitochondrial abnormalities can also activate inflammasomes and release proinflammatory factors, prompting fibroblasts in the wound microenvironment to transition to a myofibroblast phenotype, contributing to hypertrophic scar formation [16]. Additionally, dysfunction of mitochondrial metabolic enzymes can impair collagen synthesis by fibroblasts [17], ultimately resulting in hypertrophic scarring or poor healing outcomes.

Given the central role of mitochondrial dysfunction in impaired wound healing, numerous mitochondrion-targeted therapeutic strategies are under active investigation [18–23]. However, most current approaches target only a single aspect of dysfunction and lack long-term efficacy, often failing to fundamentally rescue impaired mitochondrial function. In this context, mitochondrial transfer has emerged as a promising, novel intervention. First described in 2006, mitochondrial transfer involves the intercellular transfer of intact mitochondria [24]. By improving the mitochondrial pool of the recipient cell, mitochondrial transfer fundamentally restores mitochondrial function, providing a new therapeutic paradigm for conditions associated with mitochondrial dysfunction. To date, mitochondrial transfer has been extensively investigated in the fields of ischemic stroke [25], cancer [26], cardiovascular diseases [27], idiopathic inflammatory diseases [28], and other conditions. These findings consistently indicate that mitochondrial transfer can effectively alleviate oxidative stress, improve energy metabolism, and modulate repair signaling, thereby protecting recipient cells and promoting tissue repair. Recently, the role of mitochondrial transfer in wound repair has garnered increasing attention. Preliminary evidence supports its protective role in wound healing. For instance, mitochondria from human umbilical cord mesenchymal stem cells (h-UCMSCs) significantly increase energy metabolism in recipient cells *in vitro* and promote wound healing and tissue regeneration in rat models of digestive tract fistulas [29]. Furthermore, healthy mitochondria extracted from a patient’s own skeletal muscle have been shown to accelerate the repair of chronic pressure ulcers [30].

Research on mitochondrial transfer in wound healing is currently in a phase of vigorous development; yet, a systematic review of the field is lacking. Therefore, this review focuses on the potential value of mitochondrial transfer in wound repair. Through a systematic elucidation of its mechanisms of action and a detailed exposition of the current application status of mitochondrial transplantation therapy in wound repair, it aims to provide a reference for subsequent research and clinical translation in this area.

## Review

### Physiological basis of mitochondrial transfer

#### Intrinsic mitochondrial motility

Mitochondria are not static organelles that are passively distributed throughout the cytoplasm. Instead, their precise subcellular localization is dynamically regulated through active intracellular trafficking. This system allows for the adaptation to fluctuating metabolic demands in distinct cellular zones, the response to stress, and the maintenance of overall cellular homeostasis. This sophisticated process is orchestrated by the coordinated interplay of three core components: the cytoskeletal framework, molecular motor proteins, and specific adaptor complexes. The cytoskeleton provides structural “railways” for mitochondrial movement. Microtubules and actin filaments (F-actin) serve as the primary tracks, each of which plays distinct and sometimes complementary roles, depending on the cell type and physiological context. Microtubules constitute the major highway for long-range mitochondrial transport in most mammalian cells. Their intrinsic polarity, with plus-ends typically oriented toward the cell periphery or axonal terminals and minus-ends oriented toward the centrosome or cell body, dictates the directionality of mitochondrial movement [31]. Conversely, F-actin networks primarily facilitate short-range mitochondrial repositioning or transport in specialized cell types such as myocytes and yeast, a process mediated by myosin motor proteins [32]. The molecular motors responsible for mitochondrial translocation along these tracks are predominantly members of the kinesin and dynein superfamilies. Kinesins are generally plus-end-directed motors that transport mitochondria from the perinuclear region toward the cell periphery to energize localized areas of high ATP demand, such as active growth cones or synaptic terminals. Dynein, a minus-end-directed motor complex, mediates the retrograde transport of mitochondria from peripheral regions back toward the cell center for potential quality control, recycling, or degradation. Adaptor proteins are responsible for mediating the binding between motor proteins and mitochondria, among which the Milton–mitochondrial Rho GTPase (Miro) complex represents the best-characterized adaptor module identified to date. Milton was first discovered in Drosophila and has two orthologs in mammalian cells: trafficking kinesin protein (TRAK) 1 and TRAK2. Miro proteins are localized to the outer mitochondrial membrane (OMM) and can directly bind to Milton (TRAK) proteins. Milton (TRAK) proteins interact with Miro at one end and bind to kinesin or dynein at the other, ultimately anchoring motor proteins to the mitochondrial membrane and initiating the intracellular transport of mitochondria [33].

**Figure 1:**
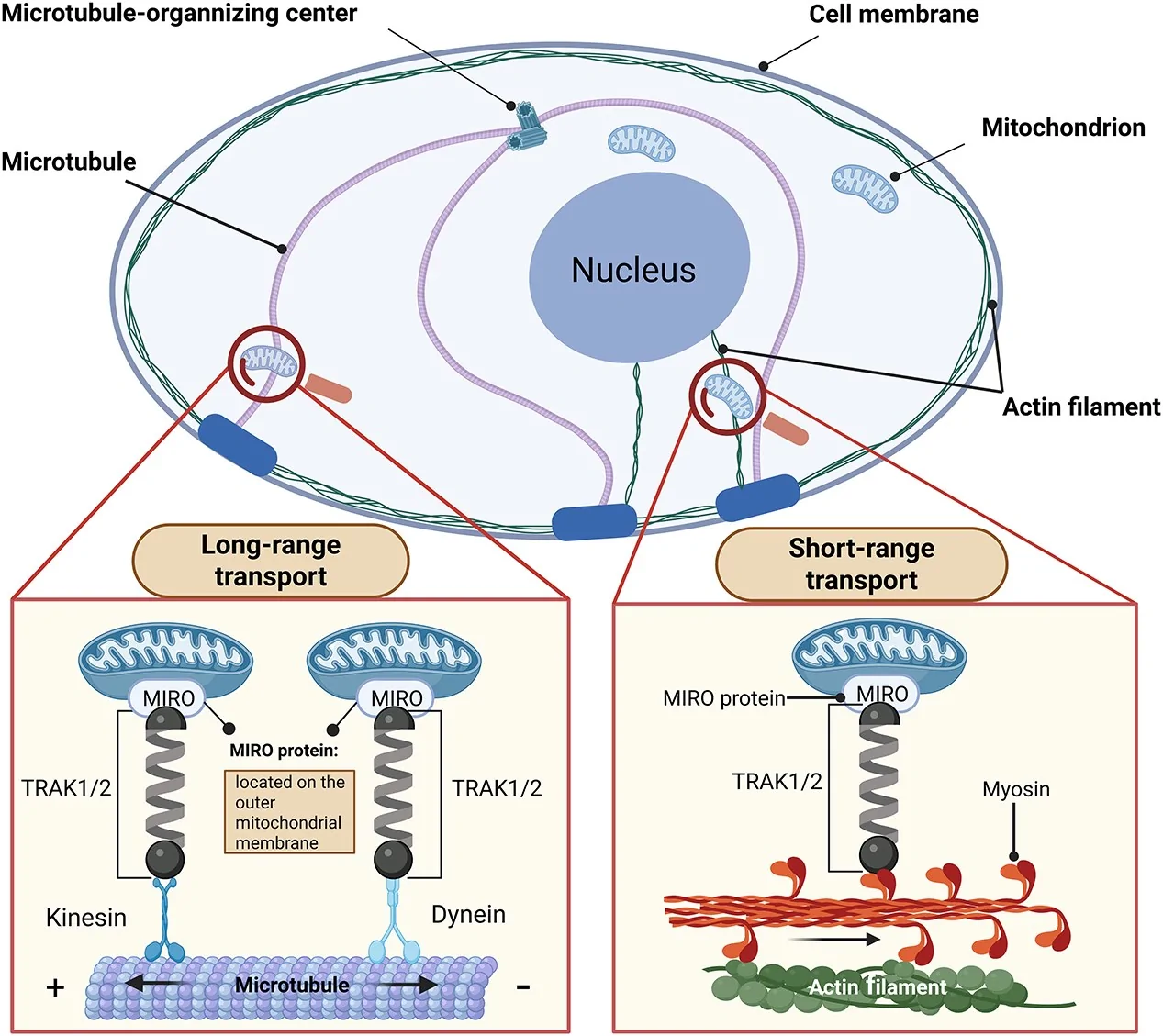
Intrinsic motility of mitochondria. *MIRO* mitochondrial Rho GTPase, *TRAK* trafficking kinesin protein. Figure was created with BioRender.com

Consequently, mitochondrial trafficking involves two complementary systems: long-range, microtubule-based transport driven by kinesin/dynein and short-range, F-actin-based transport mediated by myosin motors. These pathways work in concert to precisely distribute mitochondria throughout the entire cellular volume [34]. During long-range transport, the small GTPase-like proteins Miro1/2 first bind to one end of Milton (TRAK1/2). Meanwhile, the other end of Milton (TRAK1/2) connects to kinesin or dynein, completing the linkage between mitochondria and motor proteins. Mitochondria then move along microtubules driven by kinesin or dynein. During short-range transport, one end of Milton (TRAK1/2) still binds to Miro proteins, but the other end no longer associates with motor proteins. Instead, it binds to myosin, anchoring mitochondria to F-actin and mediating mitochondrial movement along F-actin [35] (Figure 1).

This capacity for directed intracellular motility is fundamental for cellular physiology. It is crucial for the equitable partitioning of mitochondria during cell division, establishing cellular asymmetry, and compartmentalizing subcellular functions. By ensuring precise spatial targeting, it tailors mitochondrial functional output to the specific energetic and signaling requirements of distinct cellular domains [36]. Furthermore, autonomous intracellular motility is a prerequisite for the emerging phenomenon of intercellular mitochondrial transfer. Initially, mitochondria must navigate from perinuclear clusters or other cytosolic compartments to specialized sites of cell–cell contact, such as tunneling nanotubes (TNTs), fusion interfaces, or membrane regions primed for microvesicle (MV) budding. This targeted positioning is the essential first step in launching subsequent transmembrane transfer events [37, 38]. Only when mitochondria are spatially localized at the aforementioned intercellular contact sites can the subsequent transmembrane transport process be further initiated. The orderly intercellular transfer of mitochondria cannot be achieved without an efficient and controllable intracellular motility system. In addition, intracellular mitochondrial motility and intercellular transfer share core molecular regulatory elements, such as Miro proteins, suggesting a close regulatory relationship and overlapping molecular mechanisms between the two processes.

#### Pathways of mitochondrial transfer TNTs

In 2004, Rustom and his team first described TNTs as a novel form of intercellular connection [39]. TNTs are cytoskeletal-based, tubular structures that form between cells. These unique membrane protrusions, primarily supported by F-actin, can freely span the extracellular space to connect distant cells [40]. Live-cell imaging reveals that TNTs are transient structures, which last from minutes to hours *in vitro* [39]. Their formation occurs through two mechanisms: in an actin-driven mechanism, a cell extends a pseudopodium-like protrusion containing F-actin that fuses with the edge of an adjacent cell, leading to TNT formation. In a cell displacement mechanism, two closely situated cells move in opposite directions, extending membrane protrusions that result in TNT formation. As nanoscale membrane channels between cells, TNTs range from 50 to 1500 nm in diameter and 5200 μm in length. They mediate the exchange of substances between nonadjacent cells within the same tissue, facilitating the transfer of molecules, pathogens, and organelles between cells [41].

TNTs have been confirmed as a principal pathway for mitochondrial transfer and extensively participate in mitochondrial trafficking across various tissue cell types. For instance, tumor cells acquire exogenous mitochondria *via* TNTs, which not only increase their proliferation, invasion, and metastasis but also significantly increase immune evasion, chemotherapy resistance, and the oxidative stress response. Bone marrow mesenchymal stem cells (BMSCs) transfer mitochondria to Müller cells through TNTs, remodeling their metabolic processes and suppressing glial proliferation, thereby partially preserving visual function in degenerating rat retinas. Furthermore, following ischemic stroke, mesenchymal stem cells (MSCs) upregulate the expression of Miro1 and mitofusin-2 (Mfn-2), promoting TNT formation with neurons and initiating mitochondrial transfer. This process ultimately leads to the restoration of impaired neuronal energy metabolism, a reduction in the infarct volume, and an improvement in neurological function [41–43].

##### Extracellular vesicles (EVs)

EVs are membrane-bound vesicles that are released from various cell types by budding from the plasma membrane. They can encapsulate biological macromolecules or particulate substances such as proteins, lipids, and nucleic acids and transfer these materials between cells [44]. The important role of EVs in cell communication has been widely recognized, and their involvement in mitochondrial transfer has become increasingly clear. They can encapsulate mitochondria and facilitate their transfer between cells. EV-mediated mitochondrial transfer serves two primary functions: the removal of damaged mitochondria and the transport of functional mitochondria.

In terms of the elimination of damaged mitochondria, MSCs under oxidative stress conditions are taken as an example. MSCs can regulate mitophagy through EVs, targeting autophagosomes to degrade intracellular depolarized mitochondria. Specifically, intracellular depolarized mitochondria are first loaded into vesicles containing microtubule-associated protein 1 light chain 3. These vesicles then migrate to the cell membrane and fuse into budding membrane structures, followed by release *via* MVs (0.1–1 μm in diameter) mediated by inhibitin domain-containing protein 1, ultimately excreting the mitochondria extracellularly. The released MVs can be phagocytosed by macrophages, where their encapsulated mitochondria fuse with the macrophage mitochondrial network and are repurposed, thereby increasing the bioenergetic levels of macrophages [45]. Studies of brown adipocytes also support this function. In brown adipose tissue (BAT), brown adipocytes undergoing thermogenic stress release oxidatively damaged mitochondrial components *via* mitochondrial-derived vesicles (MDVs) into the EV pathway to maintain cellular homeostasis. These EVs are subsequently cleared by BAT-resident macrophages, safeguarding normal BAT thermogenic function [46].

With respect to the transport of functional mitochondria, M1 macrophages transfer mitochondria to pancreatic β-cells *via* EVs. Upon fusion with existing mitochondria within β-cells, these transferred mitochondria induce intracellular Fe^2+^ accumulation, leading to increased mitochondrial ROS production and lipid peroxidation. This process results in mitochondrial rupture, the release of mtDNA into the cytoplasm, the activation of the cGAS–STING pathway, and ultimately, the ferroptosis of β-cells [47]. In addition to inducing cell death, exogenous mitochondria can also support recipient cell function. For instance, damaged pancreatic acinar cells internalize EVs from MSCs, acquiring functional mitochondria that reverse metabolic dysfunction and sustain ATP production [48]. Furthermore, EVs are characterized by high stability, low toxicity, and low immunogenicity [49], making them promising potential carriers for use in mitochondrial transplantation research.

##### Gap junctions (GJs)

GJs are specialized transmembrane channels composed of connexins. In the plasma membrane of individual cells, six connexins assemble to form a connexon hemichannel. Adjacent hemichannels from neighboring cells align end-to-end, forming a gap junction channel comprising 12 connexin subunits [50]. These channels serve as conduits for rapid, direct intercellular communication. Their function is regulated by the biosynthesis, trafficking, assembly, internalization, and degradation of connexins. Primarily, GJs facilitate intercellular communication, enabling the direct exchange of various substances between cells [51].

Historically, GJs were presumed to only allow the passage of molecules smaller than 1000 Da [52], such as Ca^2+^ ions and cyclic adenosine monophosphate (cAMP), while preventing the movement of proteins or nucleic acids. However, the experimental evidence now demonstrates that GJs not only mediate the exchange of small molecules between cells but also participate in intercellular mitochondrial transfer. Numerous studies have shown that GJ inhibitors and enhancers affect the quantity of mitochondrial transfer [53–55]. While this finding provides indirect evidence, it does not exclude the possibility that GJs influence mitochondrial transfer by affecting other mitochondrial transfer pathways. For instance, in a study investigating the role of mitochondrial transfer in preventing acute lung injury, gap junction channels based on connexin 43 (CX43) influenced mitochondrial transfer by mediating Ca^2+^ signaling and promoting cell adhesion and the formation of TNTs and EVs [56]. More recently, researchers utilizing immunocytochemistry, super resolution microscopy, and transmission electron microscopy have observed GJ internalization [57]. They performed immunogold labeling of CX43 in serial tissue sections and discovered that fully internalized GJs contained intact mitochondria [58], providing direct evidence for GJ-mediated mitochondrial transfer.

##### Cell fusion

Cell fusion refers to the process by which two distinct, independent cells recognize each other, adhere closely, and undergo a series of events leading to the merging of their cellular contents. This process begins with hemi-fusion, where the outer leaflet of the phospholipid bilayers of the two cell membranes merge. Subsequently, fusion pores form, allowing the cytoplasmic contents of the two cells to mix. Finally, these fusion pores expand, resulting in the complete merging of the two cells into a single entity that shares organelles and cytoplasmic components [59].

With respect to the role of cell fusion in mitochondrial transfer, reports describe the observation of permanent cell fusion during the coculture of fibroblasts with salivary gland adenoid cystic carcinoma cells lacking mtDNA and mitochondrial function. This event enables mitochondria-deficient cancer cells to immediately acquire healthy mitochondria and mtDNA, thereby restoring their mitochondrial respiration and rescuing their metabolic dysfunction [60]. However, some scholars argue that this process does not constitute mitochondrial transfer, as all cellular components merge into a single cellular entity, eliminating definable donor or recipient cells. They propose that this process is more accurately described as a form of mitochondrial vertical inheritance occurring in the opposite direction [61]. Transient partial cell fusion has also been reported. Through live-cell imaging and electron microscopy, researchers have observed two forms of partial cell fusion. One form, which is utilized to connect distant cells, comprises elongated protrusions. Unlike TNTs, these attach to the substrate rather than the cell membrane. The other form involves localized, broad, and intimate cell–cell contacts formed between adjacent cells. Cells undergoing transient partial cell fusion exhibit a focal disruption of their plasma membranes, enabling brief direct intercellular communication and the exchange of cellular materials, including mitochondria, following partial fusion [62].

##### Free mitochondria

Free mitochondria were first identified in the bloodstream. Within the circulatory system, platelets serve as a primary reservoir for mitochondria. Boudreau LH and colleagues experimentally demonstrated that stimulated platelets can redistribute mitochondria to their cell membranes before these respiring organelles are released into the extracellular milieu. These released mitochondria can then be captured and internalized by recipient cells, such as neutrophils. Transmission electron microscopy, confocal microscopy, and monoclonal antibodies against specific receptors on the OMM revealed that a portion of these released mitochondria are enclosed within EVs, whereas others exist in a free, unenveloped form [63]. In addition to platelets, adipocytes can also release mitochondria into the circulation. Assays reveal negative results for the EV markers CD63, CD9, and CD81 but positive results for the mitochondrial outer membrane protein TOM22, indicating that these mitochondria are also released in a free form into the bloodstream [64]. While exploring the characterization and origin of mitochondria in the blood of healthy mice and humans, researchers discovered that multiple lines of evidence indicate the presence of structurally intact and functionally competent mitochondria circulating in the bloodstream that originate from a variety of cellular sources. Notably, a subset of these mitochondria lacks typical cell-surface markers, suggesting that they may exist as free mitochondria [65].

**Figure 2:**
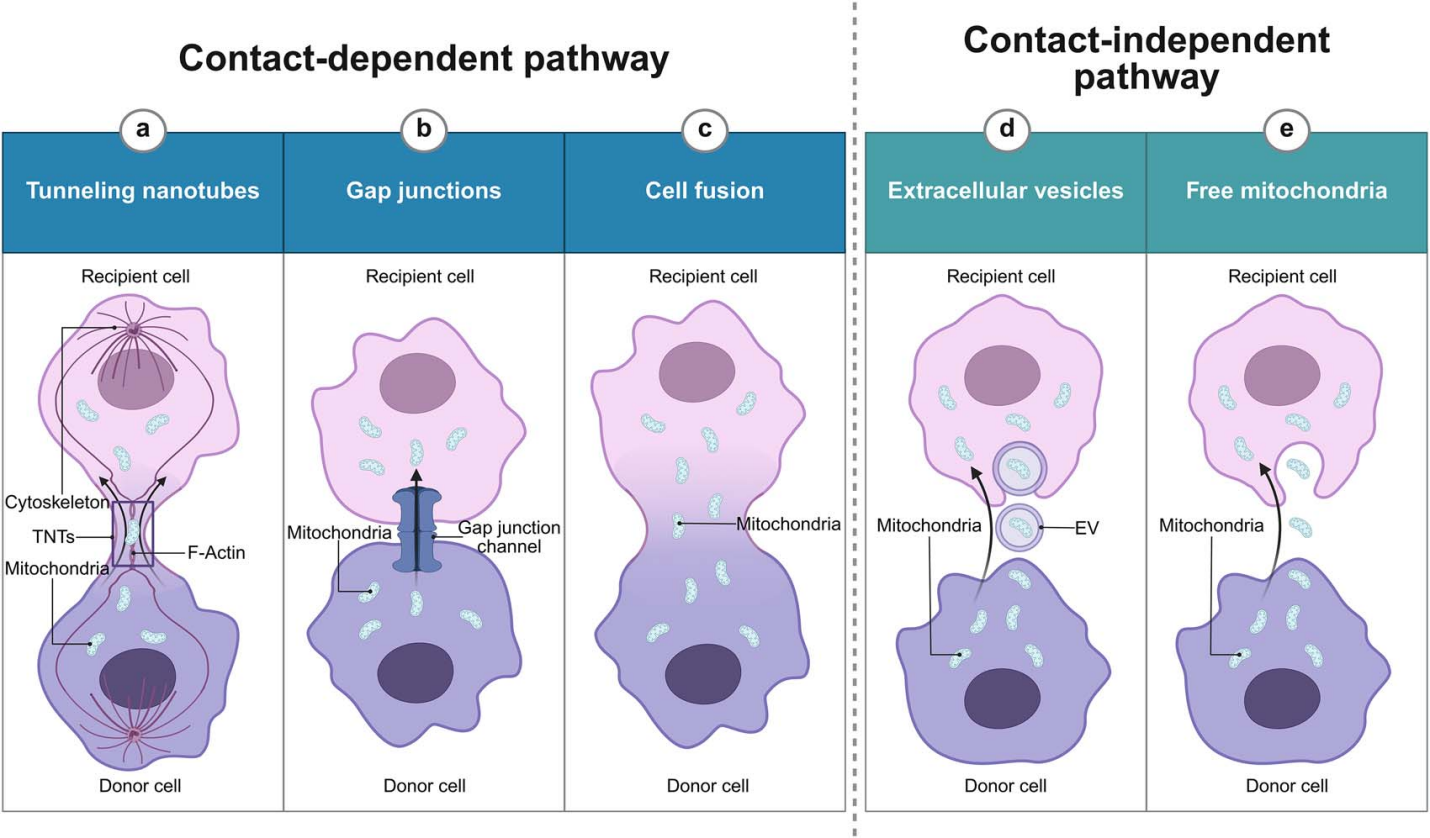
Pathways of mitochondrial transfer. *TNT* tunneling nanotubes, *F-Actin* actin filaments, *EV* extracellular vesicle. Figure was created with BioRender.com

Clark and Shay first documented the endocytosis of free mitochondria by recipient cells in 1982, coining the term “mitochondrial transformation” [66]. Some scholars have proposed that the primary uptake mechanism for free mitochondria involves macropinocytosis [67]. Additional research suggests that this endocytic process depends on the integrity of the OMM and the presence of surface fusion proteins, such as syncytin-1 and syncytin-2. These proteins may function as ligands that mediate the interaction between mitochondria and recipient cells [68] (Figure 2). Nevertheless, the precise mechanisms governing free mitochondrial uptake remain incompletely understood. A key unresolved question is why recipient cells do not activate mitophagy pathways to degrade these exogenous free mitochondria following their internalization. The underlying mechanisms remain to be elucidated.

##### Migrasome

In addition to the widely reported mitochondrial transfer pathways, migrasomes—a newly discovered cellular structure—also mediate the extracellular release of mitochondria and represent a potential novel mechanism for mitochondrial transfer. Migrasomes are specialized organelles generated by migrating cells that form on elongated tethers called retraction fibers (RFs), which trail behind motile cells. During migrasome biogenesis, specific cytoplasmic components, including organelles, can be selectively trafficked and encapsulated within these structures. As RFs disintegrate, migrasomes and their cargo are subsequently released into the extracellular milieu. The released migrasomes may be taken up by recipient cells or rupture, discharging their contents into the surrounding microenvironment [69–71]. Studies have confirmed that during mild mitochondrial stress, damaged mitochondria can be specifically transported into migrasomes and expelled by migrating cells *via* this mechanism [72]. Notably, neutrophils and macrophages have been identified as major sources of migrasomes [73]. To date, research has demonstrated that migrasomes facilitate the expulsion of damaged mitochondria, serving as a mitochondrial quality control pathway in migrating cells [74]. Whether migrasomes can mediate the intercellular transfer of healthy mitochondria remains unexplored. The physiological and pathological roles of migrasomes in mitochondrial transfer *in vivo* and whether they should be formally classified as a mitochondrial transfer pathway warrant further investigation.

The inherent intracellular motility of mitochondria provides the foundation for their intercellular transfer. Mitochondria can traverse cellular boundaries between different cells *via* multiple routes, including TNTs, EVs, GJs, cell fusion, and the release of free mitochondria. Together, these diverse transfer mechanisms establish a multidimensional and multilayered network for intercellular mitochondrial communication. Notably, mitochondrial transfer is not a spontaneous or random event. Its initiation and efficiency are tightly regulated by precise microenvironmental signals and molecular mechanisms. The following sections will systematically outline the regulatory framework governing mitochondrial transfer, focusing on the triggers that initiate this process and the key molecular players that modulate it.

### Triggers and regulation of mitochondrial transfer

#### Factors that trigger mitochondrial transfer

Mitochondrial transfer constitutes a vital compensatory mechanism activated by cells under stress or injury, fundamentally serving as a response of the donor cell to the functional demands of the recipient cell. When donor cells recognize a recipient cell in a stressed state or receive “signals” released from damaged cells, they initiate the transfer of their mitochondria to the recipient. Current research indicates that mitochondrial transfer can be triggered by multiple intersecting signals. In addition to the most frequently observed indicators of oxidative stress, factors such as compromised mitochondria and inflammatory signals play key roles in activating this process.

According to the present body of research, oxidative stress resulting from ROS accumulation is the most common trigger of mitochondrial transfer. Experiments have shown that ROS-induced oxidative stress can regulate the opening of connexin channels *via* the phosphatidylinositol 3-kinase (PI3K) signaling pathway, permitting mitochondria to be transferred from BMSCs to hematopoietic stem cells [75]. Furthermore, in acute myeloid leukemia models, accumulated ROS stimulate the transfer of mitochondria from BMSCs to leukemia blasts [76]. Additional comparative experiments between oxidative stress-induced chondrocytes and normal, unstimulated chondrocytes revealed a significant increase in mitochondrial transfer events among stressed chondrocytes [77]. Concurrently, MSCs can transfer their own mitochondria to macrophages under oxidative stress conditions, initiating a rescue response [45]. Oxidative stress-induced mitochondrial dysfunction in osteocytes similarly triggers adjacent healthy osteocytes to transfer mitochondria, compensating for the functional deficit [78].

Damaged mitochondria have also been identified as one of the triggers for mitochondrial transfer. Studies indicate that following the phagocytosis and degradation of compromised mitochondria by MSCs, antiapoptotic pathways within the MSCs are activated, initiating mitochondrial transfer from the MSCs to the recipient cells [79]. Similarly, in response to mitochondrial products and damaged mitochondria released by injured cells, MSCs facilitate repair and regeneration by transferring their mitochondria to compromised cells [80]. When a cell’s own mitochondria are damaged, jeopardizing normal physiological function and cell viability, it can actively induce the uptake of exogenous healthy mitochondria to compensate for the functional deficit [81–83]. Additionally, the activation of proinflammatory cytokines, such as tumor necrosis factor-α (TNF-α), IL-1β, and interferon-γ (IFN-γ), can effectively induce the formation of TNTs and EVs, triggering mitochondrial transfer [84, 85].

Although the current evidence confirms multiple triggers for mitochondrial transfer, with preliminary experimental support provided by related studies, the specific regulatory pathways through which these triggers mediate mitochondrial transfer remain incompletely elucidated and warrant further investigation.

#### Regulators of mitochondrial transfer

Intercellular mitochondrial transfer is a critical process for maintaining cellular homeostasis; it is involved in the repair of pathological damage and affects disease progression. Its regulatory mechanisms are complex and diverse. The core regulatory molecules involved in this process include CD38 (cluster of differentiation 38, cyclic ADP-ribose hydrolase), Miro proteins, and CX43. These factors orchestrate intercellular mitochondrial transfer *via* distinct signaling pathways and specific modes of action.

##### Regulation of mitochondrial transfer by CD38

CD38 is a type II transmembrane glycoprotein composed of a 45 kDa single polypeptide chain with multifunctional enzymatic activity. Approximately 90% of CD38 is located on the cell membrane, with the remaining 10% distributed in other organelles [86]. Studies have shown that CD38 participates in intercellular mitochondrial transfer through two primary pathways: EVs and TNTs. Mechanistically, as one of the major nicotinamide adenine dinucleotide (NAD^+^)-consuming enzymes, CD38 hydrolyzes NAD^+^ to generate products such as cyclic adenosine diphosphate ribose (cADPR) and nicotinamide, making it a critical component of NAD^+^ homeostasis [87]. cADPR acts as a vital second messenger that targets receptors on the endoplasmic reticulum, triggering the release of stored calcium ions into the cytoplasm [88]. These released Ca^2+^ ions accumulate on the inner side of the cell membrane, promoting the repolymerization of F-actin [89]. Given that F-actin is a core component of the cytoskeleton, its polymerization status directly influences EV release and TNT formation, thereby regulating mitochondrial transfer. During EV-mediated mitochondrial transfer, the upregulation of CD38 gene expression can increase the expression of inositol trisphosphate receptor (IP3R) and mitochondrial Ca^2+^ levels in MSCs, consequently increasing the release of mitochondria-containing EVs from MSCs [90].

Under pathological conditions, cerebral ischemia can upregulate CD38 expression in astrocytes. This upregulation further activates the CD38–cADPR–Ca^2+^ signaling pathway, initiating a cascade of processes, including TNT formation and the release and intercellular transport of EVs. These changes increase the transfer of mitochondria from astrocytes to compromised neurons, ultimately exerting an endogenous neuroprotective effect. Conversely, siRNA-mediated inhibition of CD38 signaling reduces mitochondrial transfer, thereby attenuating its therapeutic efficacy in mouse stroke models [81, 91]. Furthermore, CD38 is highly expressed by myeloma cells in the tumor microenvironment of multiple myeloma. The number of TNT anchor points between cells is positively correlated with CD38 expression levels in these myeloma cells, suggesting a direct association between CD38 expression and TNT attachment. The upregulation of CD38 expression significantly promotes TNT formation between myeloma cells and bone marrow stromal cells, mediating the transfer of mitochondria from stromal cells to tumor cells. This process provides OXPHOS to myeloma cells, enhancing their metabolic plasticity and proliferative potential. The inhibition of CD38 expression or treatment with CD38-blocking antibodies significantly suppresses TNT formation and intercellular mitochondrial transfer [92]. Collectively, these studies indicate that high CD38 expression promotes intercellular mitochondrial transfer, thereby increasing the functional capacity of recipient cells.

##### Regulation of mitochondrial transfer by Miro proteins

Miro proteins are mitochondrial Rho-GTPases localized to the OMM. They form nanoscale clusters along the OMM, participate in the structure of the mitochondrial contact site and cristae organizing system (MICOS) complex, and link the outer and inner mitochondrial membranes [93]. These proteins couple the MICOS with TRAK motor protein adaptors, ensuring a proper MICOS distribution within mitochondria and facilitating the coordinated delivery of both mitochondrial membranes during transport [94].

The Miro protein regulates mitochondrial transfer primarily by mediating mitochondrial transport along the cytoskeleton. As described in detail in the preceding section on mitochondrial motility, Miro facilitates this movement by forming a complex with motor proteins and adaptor proteins. This complex tethers the mitochondrion to microtubules or F-actin, enabling its translocation along these cytoskeletal tracks. This mechanism constitutes the core process for the peripheral distribution of mitochondria within the cell. Following the genetic knockout of the Miro protein, mitochondria become concentrated in the perinuclear region [95].

The regulatory role of Miro proteins in mitochondrial transfer has been substantiated through numerous experiments. For instance, Ahmad *et al.* demonstrated that Miro1 overexpression in MSCs increases mitochondrial transfer, whereas Miro1 knockdown eliminates the rescue effect of MSCs on recipient cells [96]. Similarly, a study in which cerebral ischemia–reperfusion injury was induced confirmed that Miro1 expression levels in BMSCs influence the efficiency of mitochondrial transfer. Moreover, the results of this investigation indicated that Miro1 can potentiate the activation of the Pink1–parkin mitophagy pathway. This mechanism contributes to further improving mitochondrial quality, thereby facilitating recovery from mitochondrial damage induced by ischemia–reperfusion and concurrently increasing the capacity of BMSCs to mitigate cerebral injury [97]. Additional evidence has shown that diabetes-associated damage downregulates Miro1 in MSCs, constraining their mitochondrial transfer capacity [98]. The absence of Miro proteins directly halts mitochondrial motility, resulting in a complete loss of the cellular mitochondrial transfer ability [99, 100].

##### Regulation of mitochondrial transfer by CX43

CX43, which is encoded by the Gja1 gene, belongs to the connexin family. Connexins are widely distributed in the human body, are expressed in >110 cell types, and exhibit dynamic, time-dependent expression within cells and tissues [101].

As a canonical connexin, the primary function of CX43 is to form gap junction channels, with its molecular structure and expression level directly dictating the quality and efficiency of intercellular substance transport mediated by these channels [102, 103], including mitochondrial transfer. For example, iron oxide nanoparticles (IONPs) can selectively increase mitochondrial transfer from human mesenchymal stem cells (hMSCs) to diseased cells by triggering the formation and enhancing the function of GJs containing CX43. In a mouse model of pulmonary fibrosis, hMSCs engineered with IONPs exhibited increased CX43-related intercellular mitochondrial transfer, significantly alleviating the progression of fibrosis [104]. CX43 has also been detected within TNTs mediating mitochondrial transfer [105], influencing TNT-mediated mitochondrial relocation. Studies suggest that CX43 may modulate the TNT length and number by regulating signaling pathways such as cAMP/PKA, p38, ROCK, and FAK signaling [106, 107], thereby controlling intercellular mitochondrial transfer *via* TNTs [108]. Traumatic brain injury increases the expression of functional CX43 in astrocytes after the artificial overexpression of *GJA1-20 K*, significantly increasing the transfer of healthy mitochondria from astrocytes to injured neurons *via* TNTs [109]. While other studies have not fully elucidated the specific CX43-mediated signaling pathways regulating mitochondrial transfer, their findings collectively demonstrate that CX43 deficiency markedly reduces transfer efficiency, whereas CX43 overexpression increases transfer efficiency, underscoring the central, facilitative role of CX43 [110, 111].

**Figure 3:**
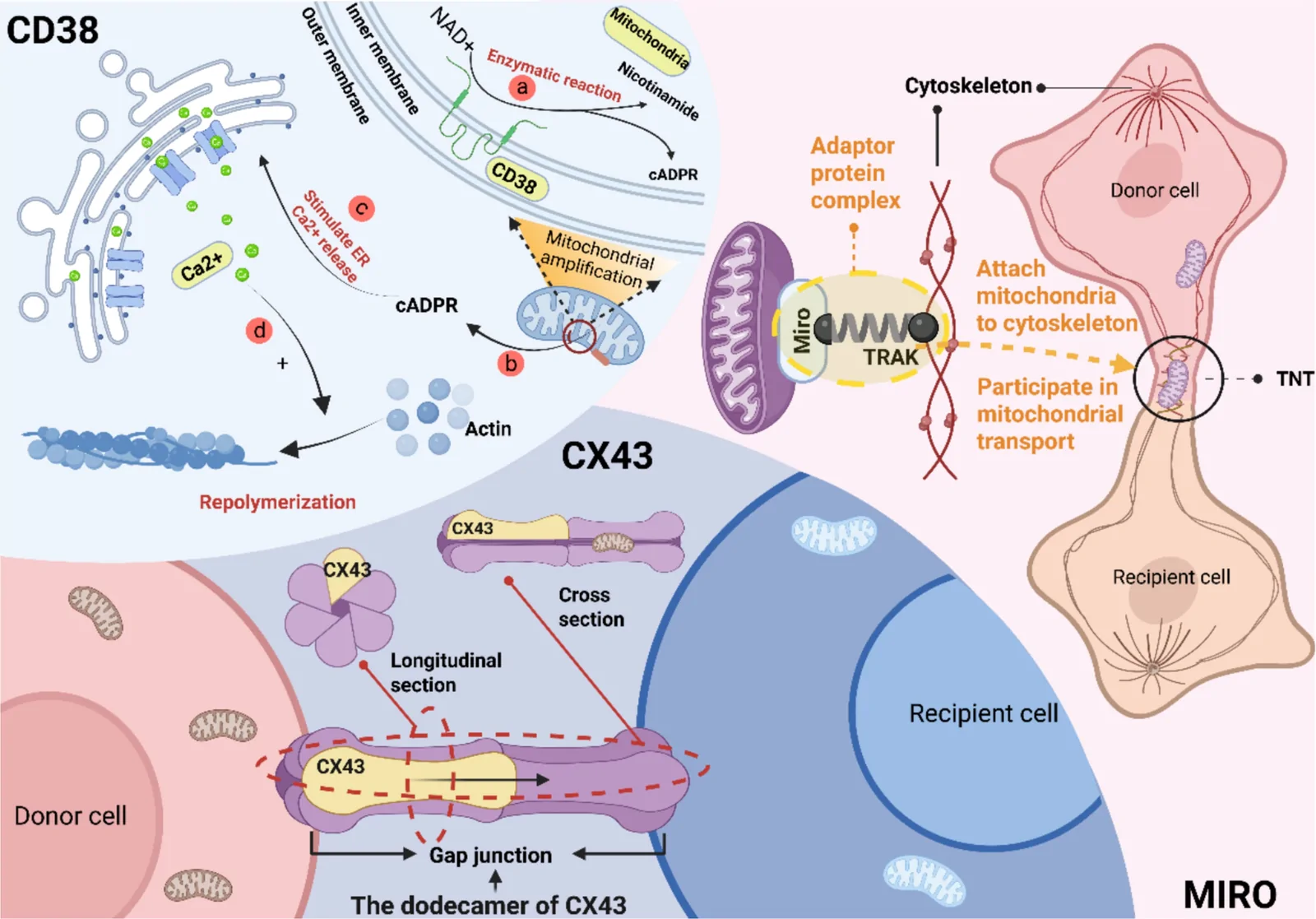
Regulation of mitochondrial Transfer. *CD38* cluster of differentiation 38, *NAD+* nicotinamide adenine dinucleotide, *cADPR* cyclic adenosine diphosphate ribose, *CX43* connexin 43, *MIRO* mitochondrial Rho GTPase, *TNT* tunneling nanotube, *TRAK* trafficking kinesin protein. Figure was created with BioRender.com

Under pathological conditions, dysregulated CX43 expression directly affects the efficacy of mitochondrial transfer between cells. Cerebral ischemia leads to the significant downregulation of CX43 expression and function in astrocytes. This process directly impairs mitochondrial transfer from astrocytes to compromised neurons, exacerbating neuronal mitochondrial dysfunction and expanding the volume of ischemic lesions. Conversely, pharmacological or genetic upregulation of CX43 expression directly restores intercellular mitochondrial transfer, thereby ameliorating aberrant energy metabolism in injured neurons and ultimately reducing the infarct size [112]. In a chondrocyte model of oxidative stress, mitochondrial transfer from hMSCs to stressed chondrocytes was significantly increased. Studies have shown that in response to oxidative stress, chondrocytes may release stress signals, such as damage-associated molecular patterns (DAMPs), which upregulate CX43 expression in MSCs to facilitate mitochondrial donation to damaged chondrocytes, although the specific signaling pathways involved remain to be verified [77]. In sickle cell disease, hemolysis-derived free heme downregulates CX43 expression in MSCs, subsequently blocking mitochondrial transfer from megakaryocytes to MSCs. This aberrant signaling disrupts metabolic reprogramming in megakaryocytes and is correlated with inadequate LYN kinase activation in platelets, culminating in disordered platelet energy metabolism and hyperactivation [113] (Figure 3).

#### Regulatory relationships between triggers and modulators

As outlined above, initiating triggers serve as upstream signals that activate mitochondrial transfer programs, whereas modulators control the precise process and efficiency of transfer *via* core pathways. Both exhibits tightly coordinated synergistic relationships. Specifically, triggers can initiate downstream signaling pathways by activating or upregulating the expression and activity of regulatory factors, thereby promoting the occurrence of mitochondrial transfer. Regulatory factors, in turn, ensure that mitochondrial transfer proceeds in an orderly and efficient manner through mechanisms such as calcium signaling and self-structure remodeling.

Emerging evidence confirms that ischemic stress activates cellular CD38, which catalyzes the production of cADPR from NAD^+^. cADPR subsequently targets endoplasmic reticulum ryanodine receptors (RyRs) and IP3R calcium channels to promote intracellular Ca^2+^ release [81]. Ca^2+^ then serves as an essential Miro cofactor. Calcium-dependent conformational changes in Miro proteins precisely regulate mitochondrial recruitment into TNTs, thereby affecting intercellular transfer [114]. As primary triggers, oxidative stress and mitochondrial damage can directly activate RyR and IP3R calcium channels on the endoplasmic reticulum, inducing substantial Ca^2+^ release into the cytoplasm [115]. In parallel, oxidative stress and mitochondrial injury directly upregulate CX43 expression, promote its phosphorylation, and shift its subcellular localization, thereby enhancing GJ-mediated communications and TNT structural stability that establishes efficient cellular conduits for organelle transfer [116]. Cumulatively, these studies converge to demonstrate that initiating triggers (e.g. oxidative stress and damaged mitochondria) operate synergistically with regulatory modulators (CD38, Miro, and CX43) to orchestrate the orderly progression of mitochondrial transfer.

Based on the analysis described above, triggering factors such as oxidative stress, dysfunctional mitochondria, and inflammatory signaling can serve as upstream initiating signals for the mitochondrial transfer process. Regulatory factors, such as CD38, Miro proteins and CX43, modulate specific aspects of this process—governing signal transduction pathways, orchestrating intracellular mitochondrial trafficking, and facilitating the establishment of intercellular junctions—thereby influencing the efficiency and specificity of transfer. These elements act in concert, playing a central regulatory role in pathological microenvironments characterized by tissue damage, oxidative stress, and inflammatory responses, ultimately determining the occurrence of mitochondrial transfer. Wound repair is a dynamic pathophysiological process encompassing the stress response, cellular recruitment, matrix remodeling, and tissue regeneration. Consequently, these triggers and regulators likely contribute to multiple critical stages of wound healing by initiating and modulating mitochondrial transfer, thereby ameliorating energy deficits in compromised cells, attenuating oxidative damage, and fine-tuning intercellular communication.

### The potential of mitochondrial transfer in wound repair

#### Alleviating oxidative stress

##### Effects of oxidative stress on wound healing

ROS are crucial regulators of wound healing and play dual roles in this process [117]. At physiological concentrations, ROS act as signaling molecules, modulating macrophage function and promoting platelet activation, angiogenesis, and re-epithelialization, thereby facilitating wound closure [117–119]. Furthermore, ROS mediate anti-infective responses in wounds by recruiting leukocytes and exerting direct bactericidal effects [120, 121]. However, excessive ROS generation leads to oxidative stress, impeding wound repair and potentially leading to chronic wounds. Mechanistically, oxidative stress directly damages keratinocytes, fibroblasts, and ECs by compromising nucleic acids, proteins, and lipids, thereby impairing their proliferation, migration, differentiation, and function [122, 123]. Concurrently, elevated ROS/RNS levels directly and indirectly promote the degradation of essential extracellular matrix (ECM) components such as collagen, proteoglycans, and hyaluronic acid, disrupting the normal process of wound repair and remodeling [124]. Moreover, oxidative stress activates proinflammatory signaling pathways, including the NF-κB and cGAS–STING pathways, leading to sustained cytokine release. This persistent inflammatory stimulus, in turn, further exacerbates ROS production, creating a vicious “oxidative stress–inflammation” cycle that ultimately inhibits the healing cascade [125].

The mitochondrial electron transport chain (ETC) is a primary endogenous source of ROS, with several generation sites (e.g. complexes II and III). Under normal physiological conditions, the ROS produced at these sites are cleared by the intracellular antioxidant defense system, maintaining cellular homeostasis. Mitochondrial dysfunction, however, can lead to ETC interruption or reduced efficiency, increasing electron leakage. These leaked electrons can directly interact with oxygen in the mitochondrial matrix, leading to elevated superoxide/hydrogen peroxide levels, disrupting the redox balance and inducing oxidative stress [3, 126, 127]. Studies have consistently demonstrated significant mitochondrial dysfunction in chronic wounds, with altered expression of multiple mitochondrial function-related genes, suggesting that oxidative stress arising from impaired mitochondrial function is a critical factor contributing to nonhealing wounds [128, 129]. Collectively, these findings indicate that damaged mitochondria are a major source of ROS that directly cause or exacerbate the oxidative stress state in chronic wounds.

##### Mitochondrial transfer alleviates oxidative stress

Research has indicated that mitochondrial transfer can directly replenish or replace dysfunctional mitochondria in compromised cells, thereby restoring cellular redox homeostasis and attenuating the detrimental effects of oxidative stress on wound repair. For instance, platelet-derived mitochondria (pl-MT) have been shown to be internalized by human dermal fibroblasts (hDFs), leading to increased respiratory chain activity, reduced ROS levels, and increased hDF proliferation and migration in scratch wound healing assays [130]. Similarly, pl-MT can be internalized by adipose-derived mesenchymal stem cells, thereby increasing their resistance to oxidative stress and increasing the viability of adipose grafts [131]. Likewise, pl-MT can be transferred to human umbilical vein endothelial cells (HUVECs), effectively lowering ROS levels, inhibiting HUVEC apoptosis, and promoting wound healing *in vivo*. Specifically, survivin has been identified as a potential key molecule that mediates the inhibitory effect of pl-MT on H_2_O_2_-induced HUVEC apoptosis [132]. Concurrently, mitochondrial transfer has been shown to significantly alleviate oxidative stress and increase the viability of skin mesenchymal stem cells (SMSCs), thereby accelerating 5-FU-induced wound healing deficits [133]. Furthermore, mitochondrial transfer has demonstrated efficacy in ameliorating oxidative stress in fibroblasts induced by UVA radiation, promoting cell proliferation and migration, and improving skin structure [134].

Notably, the antioxidant effects of mitochondrial transfer have been characterized in other tissue injury models as well. In a tendon injury model, mitochondrial transfer effectively improved mitochondrial function, reduced the levels of ROS and pro-inflammatory cytokines (IL-6 and IL-1β), and promoted cell proliferation and tissue remodeling [135]. In an osteoarthritis (OA) model, mitochondrial transfer restored mitochondrial dynamics and enhanced antioxidant system function in OA chondrocytes, ultimately significantly alleviating oxidative stress and apoptosis [136]. This cross-tissue evidence suggests that the ability of mitochondrial transfer to mitigate oxidative stress is broadly applicable, as it not only serves as a crucial mechanism for promoting cutaneous wound healing but also plays a significant role in the repair of various other tissues.

#### Attenuating inflammation

##### The effect of chronic inflammation on wound healing

Similar to ROS, inflammation exerts dual effects on wound healing. Moderate acute inflammation is essential for initiating the healing cascade, facilitating the clearance of necrotic debris and pathogens, recruiting repair cells, activating signaling pathways, and modulating the local microenvironment to support repair processes [137]. However, when inflammation becomes dysregulated, acute inflammation can transition into chronic inflammation, thereby impeding wound healing. Under chronic inflammatory conditions, persistently activated neutrophils and macrophages generate abundant matrix metalloproteinases (MMPs), leading to excessive ECM degradation and compromising the adhesion of repair cells [138–140]. Furthermore, prolonged inflammation impairs collagen deposition and hinders the phenotypic switch of macrophages from a proinflammatory (M1) state to a reparative (M2) state, resulting in insufficient granulation tissue formation and delayed epithelialization [141]. Mitochondria, which act as cellular hubs in inflammation and infection, participate in the entire “initiation–persistence–resolution” process of inflammatory responses in wounds. Mitochondrial damage results in the release of mtDNA and DAMPs, which are recognized by immune cells such as macrophages, triggering the release of proinflammatory cytokines (e.g. IL-1β and TNF-α). Moreover, aberrant mitochondrial respiratory chain function leads to excessive ROS accumulation, subsequently activating inflammatory signaling pathways such as NF-κB signaling, thereby amplifying the inflammatory response. After inflammation, mitochondrial metabolic dysregulation impedes the resolution of the inflammatory response [142–145]. These findings underscore the inseparable relationship between mitochondrial dysfunction and chronic inflammation in wounds.

##### Mitochondrial transfer alleviates inflammation

For years, targeting mitochondria has shown considerable efficacy in attenuating inflammation and promoting wound healing [146, 147]. Mitochondrial transfer, a newly discovered mechanism, can address the root cause of mitochondrial damage and has the potential to mitigate mitochondria-induced inflammation. For example, studies have shown that MSCs can transfer their own mitochondria to macrophages through TNTs and MVs, enabling macrophages to exhibit more obvious phagocytic activity and significantly enhanced antibacterial effects [148]. Similarly, another study revealed mitochondrial transfer between these two cell types. In addition to enhancing macrophage-mediated phagocytosis, researchers have reported an increased anti-inflammatory capacity in macrophages, as evidenced by the reduced secretion of proinflammatory cytokines (e.g. TNF-α and IL-8) and increased expression of the M2 macrophage marker CD206 [149]. Furthermore, mitochondrial transfer can downregulate the mRNA expression levels of the proinflammatory cytokines IL-1α and IL-1β, facilitating the rapid transition of macrophages from the M1 phenotype to the M2 phenotype and thereby reshaping the local immune microenvironment and suppressing the early inflammatory cascade [150, 151]. The levels of IL-10, an immunosuppressive and anti-inflammatory factor, in the skin are also increased after mitochondrial transfer, ameliorating inflammatory disease states [152]. In summary, mitochondrial transfer attenuates tissue inflammation and promotes tissue repair through multiple mechanisms, including modulating macrophage function, regulating inflammatory cytokine levels, and inhibiting immune cell recruitment.

#### Restoring energetic metabolism

##### The important role of energy metabolism in wound healing

The regulation of the energy supply by cellular energy metabolism directly determines the speed and quality of wound healing. Cells adapt to the distinct energy demands of various wound healing stages through metabolic reprogramming, dynamically switching between glycolysis, FAO, and OXPHOS. Glycolysis has a high reaction rate, enabling rapid ATP production to fulfill the energy demands of cells in a timely manner. FAO yields the highest ATP output (~106 ATP molecules per palmitic acid molecule), meeting the high energy needs of cells. Meanwhile, OXPHOS is both stable and efficient, providing a consistent energy supply [153–155]. Every process involved in wound repair relies on energy support, and disturbed energy metabolism constitutes one of the core mechanisms underlying the arrest of chronic wound repair. Restoring energy metabolism homeostasis and supplying sufficient energy to reparative cells promote multiple stages of wound healing. As reported in the literature for surgical incisions, ATP delivery can increase collagen synthesis, tensile strength, breaking stress, and the elasticity of wound tissue, thereby increasing the overall healing strength [156]. Mitochondria, the “powerhouses” of the cell, play a crucial role in this process. Mitochondrial dysfunction is a core disruptor of energy metabolism, leading to insufficient ATP generation. Concurrently, abnormalities in the TCA cycle within the mitochondrial matrix impede acetyl coenzyme A oxidation to cut off the “raw material supply chain” for energy metabolism and further reduce ATP production [157, 158], plunging cells into an “energy crisis”.

##### Mitochondrial transfer reconstructs energy metabolism

Multiple studies have achieved positive results for the induction of wound healing through the modulation of mitochondrial energy metabolism [12, 159, 160]. Mitochondrial transfer is a direct mechanism through which the energy metabolic network of recipient cells is reconstructed, providing crucial theoretical support for its role in accelerating wound healing.

Under conditions of oxidative stress, MSCs release EVs containing mitochondria. Macrophages internalize these vesicles, leading to a significant increase in their oxygen consumption rate (OCR) and increased bioenergetics [45]. Furthermore, starvation can also induce the cellular internalization of exogenous mitochondria. By measuring the release of NAD^+^, CD38, cADPR, and Ca^2+^, starvation was shown to promote the endocytosis of exogenous mitochondria *via* the NAD^+^–CD38–cADPR–Ca^2+^ signaling pathway. Further studies revealed that the expression of glycolysis-related genes is downregulated after mitochondrial transfer. The expression of two key rate-limiting enzymes involved in glycolysis, hexokinase (HK) and pyruvate kinase M2 (PKM2), decreases, whereas the expression of citrate synthase (CS) and isocitrate dehydrogenase 2 (IDH2), two key enzymes in the TCA cycle, increases. These changes are accompanied by the restoration of the mitochondrial membrane potential and an increase in the OCR/ECAR (extracellular acidification rate) ratio [161]. BMSCs reportedly exhibit robust mitochondrial transfer activity in coculture systems *in vitro*. Recipient cells that internalized healthy mitochondria display an increased mitochondrial respiratory capacity, improved metabolic adaptability, and increased ATP production, leading to better proliferation and migration capabilities [162, 163]. Intercellular mitochondrial transfer has also been observed in coculture systems involving other cell types, with recipient cells experiencing a 1.5-fold increase in mitochondrial ATP output following transfer [164]. Interspecies mitochondrial transfer has also been documented. The literature indicates that mitochondria from mouse muscle cells are effectively transferred into human fibroblasts (hFBs) with mtDNA mutations and that these mouse mitochondria integrate into the hFBs, thereby enhancing mitochondrial metabolic function and promoting cellular respiration to lead to bioenergetic reprogramming. The data suggest that hFBs receiving exogenous mitochondria experience varying degrees of improvement in maximal respiration, basal respiration, spare respiratory capacity (the difference in the OCR between maximal and basal respiration), and total ATP production [165]. Further studies employing confocal microscopy have shown that mitochondria-rich EVs are transferred into hypoxic-damaged recipient cells and integrate with their endogenous mitochondrial network. This process significantly increases intracellular ATP production and restores bioenergetics in recipient cells [166].

These experimental findings underscore the potential of mitochondrial transfer in reconstructing cellular metabolism, providing novel strategies for salvaging wounds with impaired energy metabolism.

#### Promoting angiogenesis

##### The role of angiogenesis in wound healing

Angiogenesis in wound healing is a multistep process initiated by hypoxic signaling and modulated by cytokines, leading to EC activation and vascular structural remodeling. The induction of hypoxia-inducible factor-1α (HIF-1α) expression in a hypoxic microenvironment is central to this process; HIF-1α drives EC proliferation, migration, and the formation of functional vascular networks through signaling pathways involving vascular endothelial growth factor (VEGF). As a critical rate-limiting step in wound repair, angiogenesis reconstructs the local blood supply, providing oxygen, nutrients, and signaling molecules to reparative cells while also clearing metabolic waste, thereby facilitating wound closure [167–169]. Conversely, aberrant angiogenesis directly leads to delayed healing, with impaired angiogenesis being a common feature of many chronic wounds [170, 171].

Notably, mitochondrial structure and function are intimately linked to angiogenesis. For instance, the expression of optic atrophy 1, a mitochondrial inner membrane fusion protein, rapidly increases in response to angiogenic stimuli to limit NF-κB signaling, thereby promoting angiogenic gene expression and angiogenesis [172]. The EC-specific loss of FUN14 domain-containing protein 1 (FUNDC1), a mitochondrial outer membrane protein, disrupts the formation of mitochondria-associated endoplasmic reticulum membranes in ECs, reduces the expression of vascular endothelial growth factor receptor 2 (VEGFR2), and impairs tube formation, spheroid sprouting, and functional vascularization [173]. Mitochondrial respiration also provides energy for angiogenesis [174]. Therefore, a disruption of mitochondrial integrity and function can impede neovascularization through various mechanisms. Restoring mitochondrial function promotes angiogenesis and enhances wound healing, and these effects are partially supported by experimental evidence [175–177].

##### Mitochondrial transfer promotes angiogenesis

Research has demonstrated that intercellular mitochondrial transfer effectively enhances mitochondrial function and biological activity in ECs, promoting angiogenesis and the maintenance of vascular homeostasis and thereby advancing tissue repair processes in pathological conditions such as ischemic injury [178]. In studies investigating mitochondrial transfer and angiogenesis, MSC-derived mitochondria are recognized as key proangiogenic mediators. Specifically, MSC-derived mitochondrial transfer can directly repair mitochondrial dysfunction in ECs. This process involves reprogramming glutathione metabolism to activate the mTORC1 pathway, leading to the upregulation of phosphorylated eukaryotic translation initiation factor 4E-binding protein 1 (p4E-BP1) and VEGFR2 expression. Ultimately, this process promotes tip cell transformation, a critical step in initiating angiogenesis [179]. Similarly, *in vitro* tube formation and spheroid sprouting assays have demonstrated that mitochondrial transfer from BMSCs to ECs significantly increases the angiogenic potential of ECs, with the Dll4–Notch1 signaling pathway playing a crucial regulatory role [180]. Furthermore, MSC-mediated mitochondrial transfer to mouse pulmonary microvascular endothelial cells (MPMECs) activates the TCA cycle and fatty acid synthesis, promoting endothelial proliferation and the release of proangiogenic factors, thereby enhancing vascular regeneration [181]. When transferred to recipient cells, EVs derived from MSCs containing mitochondria can restore the levels of angiopoietin-2 (Ang-2), a recognized mediator and biomarker of pulmonary and systemic vascular injury [84]. In addition to acting as mitochondrial donors, MSCs can internalize functional, respiratory-competent mitochondria from platelets *via* dynamin-dependent clathrin-mediated endocytosis. This transfer can activate de novo fatty acid synthesis within MSCs, further triggering the secretion of proangiogenic factors and ultimately improving the wound healing capacity through increased angiogenesis [182]. Additionally, experimental data revealed that healthy pl-MT can be transferred to MSCs, increasing their proangiogenic capacity through metabolic reprogramming and thereby accelerating wound healing [183].

Upon transfer to ECs, mitochondria from sources other than MSCs also exert proangiogenic effects. These mitochondria not only upregulate angiogenic gene expression in recipient ECs to increase their proliferation, migration, tube formation, and mitochondrial biogenesis but also restore vascular function, increase the rate and number of new blood vessels formed, and mitigate oxidative stress in ECs, thereby maintaining endothelial homeostasis and preventing endothelial dysfunction [184–187]. Importantly, studies suggest that exogenous mitochondria entering ECs not only directly enhance cellular function to promote angiogenesis [188] but also induce mitophagy and upregulate the expression of genes associated with mitochondrial biogenesis. This process improves EC bioenergetics and the overall adaptive capacity, thereby increasing the intrinsic ability of human ECs to be engrafted in ischemic tissues and promote neovascularization, particularly by increasing the angiogenic support provided by transplanted ECs. Notably, this process does not require the integration of exogenous mitochondria into the endogenous EC mitochondrial pool [189].

Collectively, this body of experimental evidence indicates that mitochondrial transfer effectively drives angiogenesis through multiple pathways, including the direct repair of EC function, modulation of key angiogenic signaling pathways, and promotion of tip cell transformation, providing crucial mechanistic insights and potential therapeutic strategies for addressing angiogenic dysfunction in wound repair.

#### Delaying cellular senescence

##### The effect of cellular senescence on wound healing

Cellular senescence in key repair cells is a primary impediment to wound healing. For instance, aged dermal fibroblasts exhibit reduced proliferation and migration potential during wound healing, a process that is characterized by diminished integrin α2β1 function and a disorganized actin cytoskeleton [190]. Senescent neutrophils display impaired debris and bacterial clearance from wound sites, leading to delayed healing and increased susceptibility to infection [191]. Senescent macrophages present compromised phagocytic activity and reduced expression of crucial growth factors such as VEGF, resulting in delayed wound debridement and decreased angiogenesis [192]. Concurrently, senescent cells secrete a senescence-associated secretory phenotype (SASP), predominantly comprising proinflammatory cytokines and chemokines, which affects nonsenescent cells and impedes the transition of wounds from the inflammatory phase to the proliferative phase [193]. Furthermore, the upregulation of cyclin-dependent kinase (CDK) inhibitors p21 and p16 in senescent cells inhibits CDK–cyclin complexes, causing cell cycle arrest in G1 phase and reducing the proliferative capacity [194].

##### Mitochondria and cellular senescence

While numerous theories exist regarding senescence, it is a complex process influenced by various factors. Recent research has indicated that mitochondrial alterations, including increased mtDNA mutations, changes in mitochondrial metabolism and mitophagy, and a disruption of mitochondrial dynamics, accompany senescence [195–197]. These findings suggest that mitochondrial dysfunction is an important mechanism underlying cellular senescence. In the free radical theory, the accumulation of mtDNA mutations is a core initiating event in cellular senescence. Specifically, because of the proximity of mtDNA to the mtROS generation site, ROS can directly attack mtDNA. Because mtDNA lacks histone protection and possesses a weak intrinsic repair mechanism, it is highly susceptible to mutation under ROS stress. The continuous accumulation of these mtDNA mutations further impairs mitochondrial function, ultimately driving cells into senescence [198]. These theories collectively suggest that mitochondrial abnormalities are key drivers of cellular senescence, suggesting that modulating mitochondrial function may delay cellular senescence and promote wound healing.

##### Mitochondrial transfer delays cellular senescence

Numerous studies have demonstrated that mitochondrial transfer can effectively restore mitochondrial function by delaying cellular senescence, thereby rescuing dysfunctional recipient cells. Specifically, this process involves improving the bioenergetics and vitality of recipient cells and reversing their senescent state. This strategy has already shown considerable therapeutic efficacy in a variety of age-related diseases [199–201].

Research has indicated that MSC-derived MV-mediated mitochondrial transfer can restore the mitochondrial respiratory function and reduce intracellular ROS levels in recipient cells. This transfer concurrently inhibits the p53/p21 and p16 senescence signaling pathways, thereby attenuating cellular senescence phenotypes. These effects promote angiogenesis and tissue remodeling, accelerating the healing of chronic wounds [202]. Furthermore, mitochondrial transfer downregulates the expression of intrinsic senescence-associated genes, such as insulin-like growth factor 1 receptor (IGF1R) and mitochondrial ribosomal protein S5 (MRPS5), while it upregulates the expression of the anti-aging gene Klotho [203]. In addition to regulating gene expression, other cellular senescence markers, such as senescence-associated β-galactosidase (β-GAL) activity, inflammatory cytokine levels, and the expression of the CDK inhibitors p21 and p16, have been shown to be downregulated through mitochondrial transfer [204]. Additionally, DNA methylation patterns are considered potential indicators of the epigenetic clock of aging. Mitochondrial transfer significantly reduces DNA methylation levels in aged rat skeletal muscle cells, suggesting that mitochondrial transfer may exert an antiaging effect by modulating epigenetic processes [205]. With respect to immune cell senescence, mitochondrial transfer can regulate senescence-associated cellular processes (such as cell signaling, ROS and DNA damage responses, metabolism and catabolism, and cell death) and downregulate the expression of senescence-associated differentially expressed proteins, thereby enhancing the function of senescent CD4^+^ T cells and improving their proliferative capacity *ex vivo* [206]. In the context of MSC senescence, mitochondrial transfer significantly reduces the percentage of β-GAL-positive cells. Furthermore, it leads to a marked decrease in the expression of the pro-apoptotic mediators p53 and p21 in recipient MSCs. This increase in the proliferative capacity and survival rate of senescent cells indicates a significant reversal of cellular aging [207]. In aged ovaries, mitochondrial transfer from younger cells not only restores the mitochondrial structure but also reactivates downstream energy pathways in aged granulosa cells, as evidenced by increased ATP levels, reduced levels of oxidative stress markers, and ultimately enhanced bioenergetics and survival, ultimately restoring the functional metabolism of recipient cells [208]. Moreover, the application of mitochondrial transfer in animal models of brain aging has shown promise. When mitochondria from young mice were transferred to the hippocampus of aged mice, overall mitochondrial function in the aged hippocampus was restored. Mechanistic studies further revealed that exogenous mitochondria were efficiently internalized by nestin-positive neural progenitor cells in the hippocampus, subsequently promoting the activation of the canonical Wnt signaling pathway and increasing neurogenesis in aged mice. These findings suggest that mitochondrial transfer may exert antiaging effects by restoring hippocampal neural progenitor cell function [209].

While direct evidence linking mitochondrial transfer to accelerated wound healing through antiaging mechanisms is still limited, its ability to delay cellular senescence in other physiological systems provides a strong theoretical foundation for its potential to positively influence wound repair by modulating the aging process.

#### Regulating collagen remodeling

##### Mitochondrial dysfunction impairs collagen remodeling during wound repair by inhibiting fibroblast function

The remodeling phase of wound repair fundamentally involves collagen restructuring, including the synthesis, crosslinking, and reorganization of the ECM to restore mechanical integrity and restore tissue function. Fibroblasts are the primary effector cells involved in this process and are responsible for synthesizing type I/III collagen fibrils and facilitating their orderly crosslinking. Concurrently, fibroblasts regulate the expression of MMPs and tissue inhibitors of metalloproteinases (TIMPs), preventing excessive collagen deposition or degradation and driving the transition of granulation tissue to mature tissue. Furthermore, fibroblasts interact with ECs and immune cells *via* paracrine signaling, and these cells collectively participate in vascular maturation and inflammation resolution to ensure the orderly progression of tissue remodeling. Therefore, the functional integrity of fibroblasts directly determines the quality of tissue repair during this phase [210, 211].

Research has indicated that impaired mitochondrial respiratory function directly leads to a significant decrease in OXPHOS efficiency and reduced ATP generation. This process not only compromises fibroblast proliferation and migration but also inhibits myofibroblast differentiation, which is marked by the downregulation of α-smooth muscle actin (α-SMA) and EDA-fibronectin expression. The subsequent deficiency in collagen matrix formation contributes to delayed wound healing [13, 14, 212]. In keloids, keloid-derived fibroblasts exhibit dysfunctional mitochondria [213], suggesting that mitochondrial abnormalities may drive aberrant fibroblast activation and excessive scar hyperplasia. Additionally, oxidative stress triggered by mitochondrial dysfunction causes widespread damage to nucleic acids, proteins, and lipids, directly impairing core fibroblast functions such as proliferation, migration, and differentiation [123]. Mitochondrial glycerol-3-phosphate dehydrogenase (mGPDH) is a key enzyme involved in mitochondrial energy metabolism. Aberrant overexpression of mGPDH in fibroblasts disrupts the SIRT1–c-Myc–TGF-β1 signaling axis, impairing ECM synthesis and exacerbating wound remodeling defects [17]. Moreover, cyclophilin D (CypD), a key regulator of the mitochondrial permeability transition pore (mPTP), is a critical molecule that is directly involved in regulating collagen secretion from fibroblasts. Its deficiency significantly reduces collagen deposition in skin tissue, directly compromising the mechanical strength of the wound bed and hindering structural maturation during remodeling [214]. Thus, mitochondrial dysfunction in fibroblasts can directly impact the remodeling stage of wound repair.

##### Mitochondrial transfer modulates collagen remodeling

**Figure 4:**
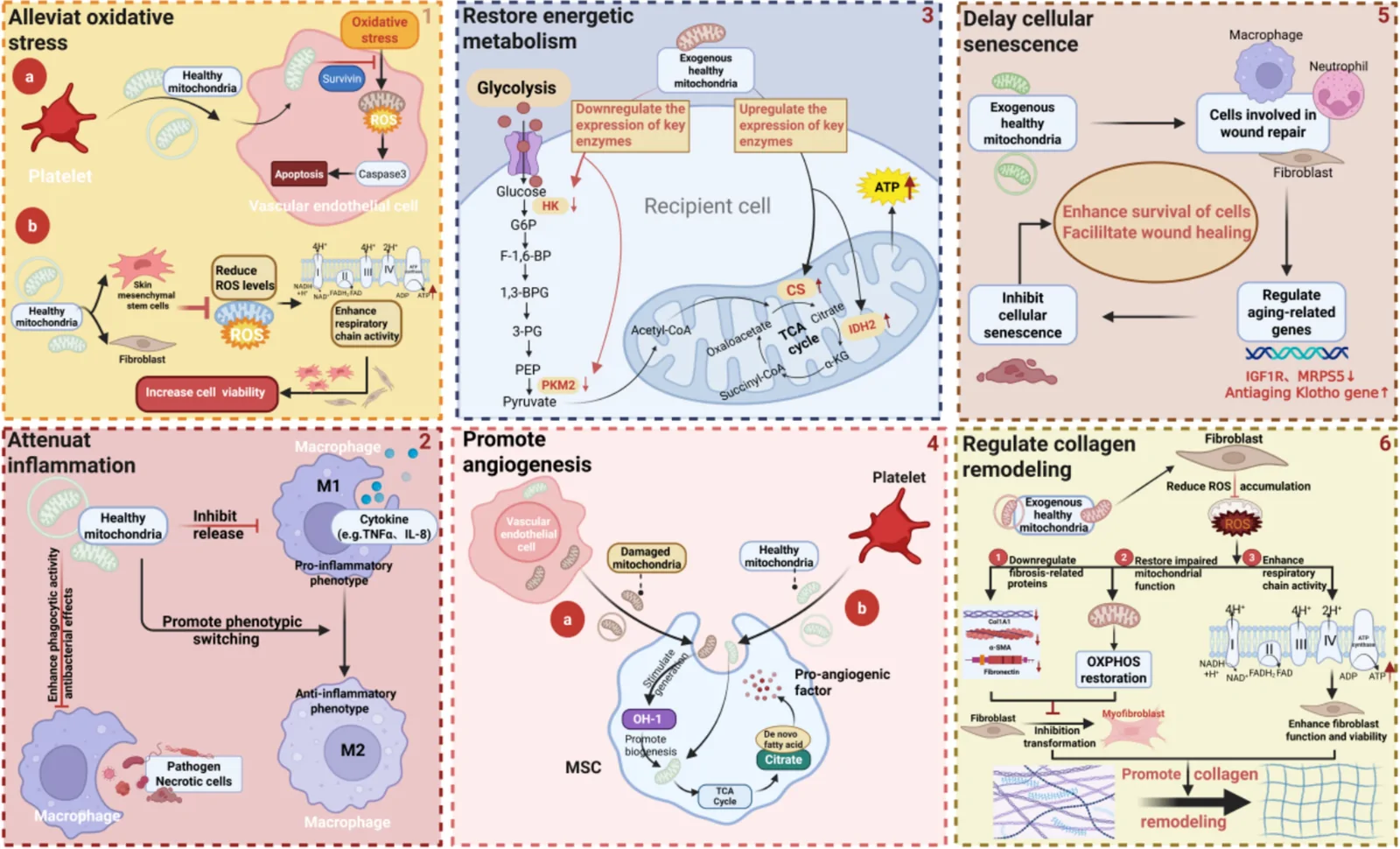
The potential mechanisms underlying mitochondrial transfer-promoted wound healing. *G6P* glucose 6-phosphate, *F-1,6-BP* fructose-1,6-bisphosphate, *1,3-BPG* 1,3-bisphosphoglycerate, *3-PGA* 3-phosphoglycerate, *PEP* phosphoenolpyruvate, *HK* hexokinase, *PKM2* pyruvate kinase M2, *CS* citrate synthase, *IDH2* isocitrate dehydrogenase 2, *CoA* coenzyme A, *α-KG* α-ketoglutarate, *TCA* tricarboxylic acid, *IGF1R* insulin-like growth factor 1 receptor, *MRPS5* mitochondrial ribosomal protein S5, *MSC* mesenchymal stem cell, *Col1A1* collagen, type1, alpha1. Figure was created with BioRender.com

Mitochondrial transfer has proven effective at rescuing fibroblast mitochondrial dysfunction and restoring fibroblast function. Studies have demonstrated that mitochondrial transfer can significantly increase respiratory chain activity in hDFs, reduce ROS levels, and promote their proliferation and migration [130]. Mitochondrial transfer also ameliorates UVA radiation-induced oxidative stress in fibroblasts and repairs the structure of damaged skin [134]. Further research has indicated that mitochondria from mouse muscle cells can be successfully transferred into and integrated within hFBs harboring mtDNA mutations, subsequently enhancing their mitochondrial function [165]. In fibrotic pathologies, transforming growth factor-β (TGF-β)-induced aberrant fibroblast activation is frequently accompanied by mitochondrial dysfunction, a key mechanism driving pathological scarring and tissue fibrosis. Transferring exogenous healthy mitochondria into TGF-β-stimulated fibroblasts effectively restores the function of impaired mitochondria, restores OXPHOS levels, reduces ROS generation, and reverses their glycolytic metabolic dependency. This activity inhibits myofibroblast transdifferentiation, reduces ECM deposition, and attenuates abnormal cell proliferation and migration, ultimately counteracting the profibrotic fibroblast phenotype [215]. Similarly, exogenous mitochondria (PN-101) derived from h-UCMSCs can be successfully transferred into TGF-β-stimulated lung fibroblasts, restoring cellular ATP levels and the mitochondrial membrane potential. Furthermore, internalized PN-101 downregulates the expression of fibrotic markers such as collagen, type 1, alpha1 (Col1A1), α-SMA, and fibronectin; inhibits the fibroblast-to-myofibroblast transition; and reduces ECM deposition, thereby antagonizing the profibrotic phenotype in lung fibroblasts [216] (Table 1). Collectively, these findings support the inference that mitochondrial transfer regulates the collagen remodeling process during wound repair by restoring mitochondrial function in fibroblasts and improving their biological activity (Figure 4).

**Table 1:** Summary of the core mechanisms of mitochondrial transfer regulating wound repair

Core effect	Mitochondrial source	Recipient cell/tissue	Mechanism	Wound healing phase
Alleviate oxidative stress	Human platelet [130]	hDFs	Enhance mitochondrial respiratory chain Attenuate ROS level	Full participation, with the inflammatory phase as the core
	Human platelet [131]	Human adipose-derived mesenchymal stem cell	Enhance antioxidant stress capacity	
	Human platelet [132]	HUVEC	Attenuate ROS level Inhibit apoptosis	
	h-UCMSC [133]	Murine SMSC	Attenuate ROS level Enhance cellular vitality	
	Rat BMSC [134]	Murine fibroblast	Reconstruct the oxidative microenvironment Promote proliferation and migration	
	Rat BMSC [135]	Rat tenocyte	Attenuate ROS level Promote proliferation	
	h-UCMSC [136]	Chondrocytes in human osteoarthritic cartilage	Enhance antioxidant system function	
Attenuate inflammation	Human BMSC [148]	Human macrophage	Enhance phagocytic activity and antibacterial effect	Primarily during the inflammatory phase
	Human BMSC [149]	Human macrophage	Enhance phagocytic activity Promote a shift toward an anti-inflammatory phenotype Inhibit inflammatory cytokine secretion	
	Testicular interstitial stem cell [150]	Macrophage	Inhibit pro-inflammatory factors secretion Promote a shift toward an anti-inflammatory phenotype	
	Rat MSC [151]	Macrophage	Promote a shift toward an anti-inflammatory phenotype	
	Murine platelet [152]	Murine macrophage	Promote a shift toward an anti-inflammatory phenotype Elevate IL-10 level	
Restore energetic metabolism	Human MSC [45]	Murine macrophage	Enhance OCR	Full participation, with the proliferative phase as the core
	Human astrocyte [161]	U87 human glioma cells	Downregulate HK and PKM2 expression Upregulate CS and IDH2 expression Increase the OCR/ECAR ratio	
	Rat BMSC [162]	Rat BMSC	Enhance OXPHOS activity Augment ATP generation	
	BMSC [163]	CD8^+^T cell	Enhance mitochondrial respiration and reserve respiratory capacity	
	Murine BMSC [164]	Human acute myeloid leukemia cell	Augment ATP generation	
	Murine myocyte [165]	hFB with mtDNA mutations	Enhance maximal, basal, and reserve respiratory capacity Augment ATP generation	
	Induced pluripotent stem cell-derived cardiomyocytes (iCMs) [166]	iCMs with Hypoxia-induced damage	Restore ATP generation	
Promote angiogenesis	h-UCMSC [179]	HUVEC	Reprogramming glutamine metabolism to activate the mTORC1 pathway Upregulate p4E-BP1 and VEGFR2 expression Promote tip cell transformation	Full participation, with the proliferative phase as the core
	Human BMSC [180]	HUVEC	Increase the number and length of lumen formation	
	Murine BMSC [181]	MPMEC	Activate the TCA cycle and fatty acid synthesis Enhance cellular proliferation Promote the release of pro-angiogenic factors	
	Human BMSC [84]	Human pulmonary microvascular endothelial cell, human small airway epithelial cell	Restore Ang-2 level	
	Human platelet [182]	Human BMSC	Activate de novo fatty acid synthesis Promote pro-angiogenic factor secretion	
	Platelet [183]	MSC	Induce metabolic reprogramming characterized by increased fatty acid synthesis Promote pro-angiogenic factors secretion	
	Murine adipose-derived stem cells [184]	HUVEC	Activate the AMPKα/PGC-1α/TFAM signaling pathway Induce mitochondrial biogenesis Enhance angiogenesis capacity	
	Not mentioned [185]	HUVEC	Upregulate HIF-1α and VEGF expression	
	Murine astrocyte [186]	Murine cerebral microvascular EC	Maintain cellular homeostasis Promote cell migration	
	Murine osteocyte [187]	Murine EC	Promote cell proliferation and migration Enhance capillary tube formation	
	Placenta-Derived MSCs [188]	EC	Upregulate VEGFR2 expression	
Delay cellular senescence	Rat MSC [202]	Rat dermal fibroblast, HUVEC	Inhibit the p53/p21 and p16 senescence signaling pathways	Full participation, with the proliferative phase as the core
	Young mouse liver [203]	Dorsal murine skin	Downregulate IGF1R and MRPS5 expression Upregulate the anti-aging Klotho gene expression	
	h-UCMSC [204]	Senescent ARPE-19 cells	Reduce β-GAL activity Downregulated p21 and p16 expression	
	Rat quadriceps femoris [205]	Skeletal muscle cells of aged rats	Reduce DNA methylation level	
	Murine fibroblast, hFB [206]	Senescent murine CD4^+^ T cells, Senescent human CD4^+^ T cells	Modulate the cellular processes associated with aging Downregulate the expression of aging-related differentially expressed proteins	
	Rat BMSC [162]	Rat BMSC	Reduce the percentage of β-GAL positive cells	
	Equine ASC [207]	Metabolism-impaired ASC	Downregulate p53 and p21experssion Enhance the proliferative capacity of senescent cells	
	Young human ovarian granulosa cell [208]	Senescent human ovarian granulosa cell	Functional metabolic rejuvenation of cell	
	Young murine liver [209]	Murine hippocampal neural progenitor cell	Activate the Wnt signaling pathway Promote cell proliferation Stimulate neurogenesis	
Regulate collagen remodeling	Human platelet [130]	hDFs	Enhance mitochondrial respiratory chain activity Reduce ROS levels Promote cell proliferation and migration	Full participation, with the remodeling phase as the core
	Rat BMSC [134]	Murine fibroblast	Promote cell proliferation and migration Repair damaged skin structures	
	Murine myocyte [165]	hFB with mtDNA mutation	Enhance cellular function	
	Murine liver [215]	TGF-β-stimulated human lung fibroblast	Repair mitochondrial dysfunction Inhibit fibroblast-myofibroblast transdifferentiation Reduce ECM deposition	
	h-UCMSC [216]	TGF-β-stimulated human lung fibroblast	Downregulate Col1A1, α-SMA and fibronectin expression Inhibit fibroblast-myofibroblast transdifferentiation Reduce ECM deposition	

*BMSC* bone marrow mesenchymal stem cell, *hDFs* human dermal fibroblasts, *HUVEC* human umbilical vein endothelial cell SMSC, skin mesenchymal stem cell, *ROS* reactive oxygen species, *MSC* mesenchymal stem cell, *OCR* oxygen consumption rate, *HK* hexokinase, *PKM2* pyruvate kinase M2, *CS* citrate synthase, *IDH2* isocitrate dehydrogenase 2, *ECAR* extracellular acidification rate, *OXPHOS* oxidative phosphorylation, *hFB* human fibroblast, *mtDNA* mitochondrial DNA, *MPMEC* mouse pulmonary microvascular endothelial cell, p4E-BP1 phosphorylated eukaryotic translation initiation factor 4E-binding protein 1, *VEGFR2* vascular endothelial growth factor receptor 2, *TCA* tricarboxylic acid, *Ang-2* angiopoietin-2, *MSC* mesenchymal stem cell, *EC* endothelial cell, *HIF-1α* hypoxia-inducible factor-1α, *VEGF* vascular endothelial growth factor, *IGF1R* insulin-like growth factor 1 receptor, *MRPS5* mitochondrial ribosomal protein S5, *β-GAL* β-galactosidase, *ECM* extracellular matrix, *Col1A1* collagen, type1, alpha1, *iCMs* induced pluripotent stem cell-derived cardiomyocytes, *ASC* adipose-derived stromal cell

Building upon the preceding evidence, mitochondrial transfer serves as a novel regulatory mechanism in wound repair, operating through multidimensional and coordinated modes of action. It exerts pivotal regulatory effects on the three core phases of wound healing—inflammation, proliferation, and remodeling—thereby providing essential support for the orderly repair of injured tissue. During the inflammatory phase, exogenous mitochondria function by increasing the activity of respiratory chain complexes and strengthening the activity of the antioxidant defense system within recipient cells. This action effectively sequesters excess ROS, ameliorating oxidative stress-induced damage. Concurrently, it modulates the balance of macrophage polarization, prompting a shift from the proinflammatory M1 phenotype to the reparative M2 phenotype, and fine-tunes the expression levels of cytokines. It suppresses the excessive levels of proinflammatory factors such as TNF-α and IL-6 while increasing the levels of anti-inflammatory factors such as IL-10, thereby curbing excessive immune cell recruitment, attenuating pathological inflammation, and establishing a stable microenvironment conducive to subsequent repair processes. In the proliferative phase, mitochondrial transfer enhances the function of the cellular respiratory chain by upregulating the expression and activity of key aerobic respiration enzymes, including CS and IDH2. This optimization of energy metabolism provides substantial ATP support to meet the bioenergetic demands of proliferating and migrating repair cells, sustaining their normal biological functions. Furthermore, exogenous mitochondria upregulate the expression of VEGFR2 in ECs while improving their mitochondrial function and biological activity. This process significantly increases EC proliferation, migration, and tube-forming capacity, accelerating wound angiogenesis and reestablishing the blood supply and nutrient delivery. During the tissue remodeling phase, mitochondrial transfer acts by repairing impaired mitochondrial function in fibroblasts, controlling oxidative stress, and counteracting abnormal metabolic reprogramming. It precisely regulates fibroblast proliferation, migration, and activation phenotypes, ensuring the proper synthesis of collagen fibers and facilitating their orderly cross-linking. Moreover, effective control of the preceding inflammatory response is crucial, as it prevents the healing process from stalling in a chronic inflammatory state, thereby promoting progression into subsequent phases and facilitating collagen network reorganization and the recovery of tissue mechanical strength. These mechanisms operate in a sequential and synergistic manner, collectively driving the orderly repair continuum from injury initiation and inflammation resolution through proliferation and tissue maturation.

Notably, direct mechanistic studies specifically linking mitochondrial transfer to skin wound healing remain relatively limited, with some inferences and evidence derived from other tissue injury models. Nonetheless, while not all studies originate from skin wound systems, the core biological processes—intercellular mitochondrial transfer, restoration of mitochondrial function, maintenance of redox homeostasis, and metabolic reprogramming—exhibit a degree of conservation and offer valuable insights across different tissue injury contexts. They therefore provide a significant theoretical framework for understanding skin wound repair.

### Mitochondrial transplantation in wound repair

Mitochondrial transplantation, an emerging cellular functional intervention technique rooted in mitochondrial transfer, involves the isolation of “high-quality” mitochondria from healthy somatic cells. These mitochondria are then infused into impaired or damaged areas of a patient’s tissue or cells, where they serve to replace “defective” mitochondria, thereby rescuing compromised cells and restoring organ function. Distinguishing this technology from mitochondrial replacement therapy (MRT) is crucial. MRT is an *in vitro* fertilization procedure designed to correct or reduce the heterogeneity of pathogenic mtDNA mutations in offspring. In contrast, mitochondrial transplantation is a postnatal therapeutic strategy that employs “high-quality” mitochondria to treat diseases [61]. Building upon the established potential of mitochondrial transfer in promoting wound healing, mitochondrial transplantation, a derivative technology, holds considerable promise for wound management, offering new avenues for the repair of chronic, refractory wounds [217–222]. Current research into mitochondrial transplantation explores two primary application modalities: injection and topical application.

#### Delivery of mitochondria *via* injection

The injection method can be further divided into local wound injection and intravenous administration. Local injection enables the direct delivery of isolated healthy mitochondria to the target site, establishing it as the current mainstream option due to its broad applicability. In the context of burn wound healing, researchers have observed a 40% increase in the OXPHOS activity of fibroblasts at the wound edge within 48 hours of the local injection of placenta-derived mitochondria into the wound. Furthermore, compared with the control treatment, the subcutaneous injection of mitochondria into burn model mice for three consecutive days resulted in significantly reduced inflammatory responses, accelerated cell proliferation, and diminished apoptosis and scar formation. These treatments also led to markedly improved wound healing rates without adverse effects such as edema or wound expansion [223]. Similarly, s subconjunctival injection of isolated mitochondria has shown significant promise in promoting the repair of acute corneal burns in mice [224]. Additional experiments have confirmed that locally injected exogenous mitochondria can effectively reach damaged epithelial tissues, modulating local inflammation, inhibiting cell death, and restoring ECM collagen components, thereby facilitating regenerative healing [225].

Regarding intravenous injection, researchers have administered mitochondria *via* the tail vein in mice and observed beneficial effects, including reduced systemic inflammation and increased bacterial clearance [226]. While intravenous delivery enables systemic distribution, its application remains limited because of particle size constraints. For instance, Zhu *et al.* observed the significant accumulation of intravenously administered mitochondria in pulmonary arteries but minimal detection in the kidneys, liver, and spleen of rats, suggesting that size constraints prevent mitochondria from crossing peripheral tissue barriers [227].

To date, injectable forms of mitochondrial transplantation have been progressively translated into clinical trials. Pediatric patients who received autologous mitochondrial transplantation at injury sites exhibited accelerated tissue repair, functional improvement, and no short-term adverse events [228]. Similarly, the injection of mitochondria isolated from healthy skeletal muscle into chronic pressure ulcers resulted in accelerated wound healing [30] (Table 2).

**Table 2:** Summary of preclinical and clinical studies of mitochondrial transplantation in large animal and human tissue injury repair

Species	Type of tissue injury	Mitochondrial source	Delivery method	Primary outcome measures
Swine	Myocardial ischemia–reperfusion injury [217]	Autologous skeletal muscle	Intracoronary perfusion	Reduced infarct size, improved cardiac function
	Acute renal ischemia–reperfusion injury [218]	Autologous skeletal muscle	Intrarenal artery injection	Decreased creatinine/blood urea nitrogen
	Isolated lung ischemia–reperfusion [219]	Autologous skeletal muscle	*Ex vivo* lung perfusion	Improved oxygenation, reduced airway pressure
Human	Myocardial ischemia [228]	Autologous skeletal muscle	Intracoronary/intramyocardial injection	Increased LVEF, improved cardiac function
	Acute myocardial infarction [220]	Autologous platelets	Intracoronary injection	Increased LVEF, improved cardiac function
	Chronic pressure wound [30]	Autologous	Local wound injection	Promoted pressure ulcer healing, improved local blood supply, reduced inflammation
	Acute cerebral ischemia [221]	Autologous skeletal muscle	Endovascular infusion (microcatheter)	No obvious adverse events, improved repair of ischemic brain tissue
Human	Idiopathic inflammatory myopathy [222]	h-UCMSC	Intravenous injection	Reduced muscle inflammation, improved muscle function
	mtDNA mutation (NCT04113447)	Not mentioned	Intracytoplasmic sperm injection	Neurological development, neonatal assessment
	Pearson syndrome (NCT06017869)	Allogeneic placenta-derived mitochondria from healthy donors	MNV-201	Treatment-related adverse events
Human	Refractory polymyositis or dermatomyositis (NCT04976140)	Allogeneic h-UCMSC	Intravenous injection	Toxicity and efficacy
	Myelodysplastic syndrome (NCT06465160)	Allogeneic placenta-derived mitochondria from healthy donors	MNV-201	Treatment-related adverse events
	Mitochondrial disease (NCT04548843)	Allogeneic placenta-derived mitochondria from healthy donors	MNV-201	Treatment-related adverse events

*LVEF* left ventricular ejection fraction, *mtDNA* mitochondrial DNA, *h-UCMSC* human umbilical cord mesenchymal stem cell

#### Delivery of mitochondria *via* topical application

In the context of topical application, the foundational approach involves diluting isolated mitochondria in a buffer solution and then applying them quantitatively to wound sites. Subsequent tissue evaluations confirmed that exogenous mitochondria are successfully internalized by wound cells. This internalization promotes cellular proliferation and migration, thereby accelerating re-epithelialization [229]. Building upon these findings, research teams have leveraged the properties of mitochondrial transfer to develop novel therapeutic materials for wound repair, expanding the applications of topical formulations.

Hydrogels containing mitochondria-enriched EVs derived from stem cells have been combined with microneedles. This combination facilitates the release of EVs and increases their uptake by the tissue. During application, this novel hydrogel microneedle patch gradually releases the encapsulated EVs, which then delivers their contained mitochondria to the tissue cells at the wound site, promoting wound healing. *In vitro* studies indicate that the EVs in this patch can be absorbed by damaged macrophages, keratinocytes, fibroblasts, and vascular ECs, improving cellular metabolic function through mitochondrial transfer. *In vivo* experiments have shown that this hydrogel microneedle patch can ameliorate metabolic dysregulation in X-ray-induced wound tissues and exerts significant therapeutic effects on radiation-induced chronic wounds [230]. Similarly, researchers have developed enucleated MSC-derived MVs containing functional mitochondria (Mito@euMVs) to address cellular senescence in chronic wounds. Their small, micrometer-scale size enables the efficient delivery of mitochondria to fibroblasts and HUVECs, thereby inhibiting and reversing high-glucose-induced cellular senescence. Researchers have integrated Mito@euMVs with a soluble PVA microneedle patch for transdermal delivery to increase their clinical applicability. In diabetic rats with pressure ulcers, this formulation resulted in a notable acceleration of the decrease in the wound area [202]. Furthermore, a crosslinked gelatin hydrogel loaded with MSCs was developed and exhibited excellent hemostatic properties. It increases mitochondrial fusion through mitochondrial transfer, thereby ameliorating mitochondrial damage and reducing inflammation and oxidative stress [231]. Another team has engineered a thermogelling, erodible hydrogel system for the *in situ* topical delivery of live mitochondria. Within 20 minutes at 37°C, this system releases up to 70% of its loaded mitochondria, demonstrating promising efficacy in tissue repair [232].

### Challenges of Mitochondrial Transplantation in Clinical Translation

Despite the emergence of mitochondrial transplantation as a research focal point, its clinical translation is hampered by substantial limitations. The evaluation of its therapeutic efficacy is currently confined to a few specific conditions, such as idiopathic inflammatory myopathies [222], ischemic heart disease [220, 228], and single large-scale mtDNA deletion syndrome [233], with clinical trials in these areas often suffering from a lack of control groups and small sample sizes, placing the field in an early exploratory phase. In the field of wound healing, while numerous studies have reported positive outcomes from mitochondrial transplantation, research remains largely preclinical, with a considerable distance from clinical trials and many fundamental questions unresolved.

#### Concerns about the biological stability of transplanted mitochondria

The wound microenvironment is rich in MMPs, especially in chronic wounds, where MMPs can be uncontrollably overactivated, impeding healing. In this environment, the stability of naked mitochondria is substantially compromised. MMPs degrade mitochondrial heat shock protein 60 (HSP60) and CX43, cleave the mitochondrial outer membrane fusion protein Mfn-2, disrupt mitochondrial membrane integrity, trigger mPTP opening, and severely impair mitochondrial structure and function [234–236]. Additionally, mitochondria are highly permeable to Ca^2+^, and exposure to micromolar concentrations of Ca^2+^ can induce mitochondrial Ca^2+^ overload, promoting mPTP formation on the inner membrane. If naked mitochondria are transplanted *via* injection, they will be exposed to high Ca^2+^ concentrations in blood (approximately 1.8 mM) or interstitial extracellular fluid, leading to mitochondrial damage [237]. Therefore, naked mitochondria are not suitable for routine use in mitochondrial transplantation therapy for wound repair.

In contrast, experimental studies using EVs to encapsulate mitochondria have shown promise. A local injection of mitochondrial-rich umbilical cord mesenchymal stem cell-derived apoptotic vesicles (UMSC-apoVs) in mouse wounds significantly increased wound closure rates [133]. Another experiment directly compared the tissue repair efficacy of naked mitochondria with that of mitochondria encapsulated in gelated MVs. The results demonstrated that this delivery system not only protected mitochondria from protease-mediated and oxidative stress-mediated damage in ischemic microenvironments *via* a physical barrier but also enhanced targeted mitochondrial uptake by recipient cells through surface modifications. Consequently, the therapeutic effects of the gelated MV delivery system were far superior to those of naked mitochondria [238]. Furthermore, comparisons between naked mitochondria and mitochondria wrapped with artificial cationic lipid membranes revealed that the encapsulated mitochondria retained better mitochondrial membrane potential and protein integrity and exhibited higher uptake efficiency by recipient cells [239].

In summary, compared with the direct use of naked mitochondria, mitochondrial delivery systems based on encapsulation technology can significantly increase mitochondrial stability and prevent structural and functional damage. When combined with other optimization strategies, they can further improve mitochondrial internalization efficiency, enabling targeted delivery and enhanced therapeutic outcomes.

#### Immunogenicity issues with transplanted mitochondria

Mitochondrial transplantation inevitably involves donor selection. The immune response elicited by allogeneic or xenogeneic mitochondria directly influences the choice between autologous and allogeneic sources and affects the clinical translation and broader application of mitochondrial transplantation therapy. Researchers isolated mitochondria from cells (primarily kidney cells) or tissues (mainly liver tissue) from 13 different species, including monkeys, cows, dogs, frogs, and lizards, and cocultured these mitochondria with diseased or normal cells (or administered them *via* a tail vein injection in mouse models). Confocal imaging was subsequently used to assess mitochondrial internalization, and systematic measurements of cell viability, ROS levels, inflammatory factor levels, metabolic indicators, animal motor function, organ function, and protein expression in tissues were performed. The results clearly indicated that neither allogeneic nor xenogeneic mitochondrial transplantation triggered significant immune–inflammatory responses initially, nor did they induce the strong rejection reactions commonly observed in traditional organ or cell transplantation models. Only under extreme conditions, such as extensive mitochondrial damage, contamination with cellular debris, or high mismatch between donor and recipient mtDNA sequences, was weak activation of innate immunity and inflammation observed, and this response was quickly resolved by the host’s immune surveillance system without compromising mitochondrial function [240]. These findings provide crucial experimental evidence for the safety of allogeneic and cross-species mitochondrial transplantation, suggesting that compared with traditional cell and organ transplants, transplanted mitochondria exhibit significantly lower immunogenicity.

However, contradictory evidence indicates that allogeneic mitochondria may induce significant inflammatory responses that are potentially linked to mitochondria-derived DAMPs, causing allograft dysfunction [241]. Some experimental results suggest that extruded free mitochondria can also act as unique danger signals, triggering inflammation [242]. Mitochondria released from necroptotic cells are phagocytosed by human macrophages and dendritic cells, leading to dysregulated cytokine production in macrophages and the induction of dendritic cell maturation [243]. Extracellular mitochondria can also activate ECs, increasing cytokine and chemokine production and increasing the risk of ectopic immune-mediated transplant rejection [244]. Furthermore, Boudreau *et al.* demonstrated that the intravenous injection of extracellular mitochondria in mice promoted neutrophil adhesion to vessel walls, activated neutrophils and provoked an inflammatory response [63]. Similarly, Zhang *et al.* showed that extracellular mitochondria can activate neutrophils, resulting in immunostimulatory effects [245].

These conflicting findings present serious challenges to the safety of mitochondrial transplantation. Moreover, during wound repair, the damaged skin microenvironment, which is characterized by the enrichment of inflammatory factors, infiltration of large numbers of immune cells (e.g. macrophages and neutrophils), a compromised skin barrier, and microbial dysbiosis [246, 247], further complicates the application of mitochondrial transplantation therapy. Existing studies have not yet systematically analyzed or experimentally investigated the immune response risks of mitochondrial transplantation within the complex microenvironment of damaged skin. Therefore, the potential immune reactions associated with mitochondrial transplantation in wound repair require further investigation.

Notably, the use of exosomes as natural vesicle carriers has inherent advantages, such as low immunogenicity and nononcogenicity. They can encapsulate mitochondria to form a stable delivery system, preventing direct exposure of free mitochondria to the recipient immune system and reducing the release of immunostimulatory molecules such as mtDNA and DAMPs, thereby lowering the probability of activating innate immune pathways. Simultaneously, they protect mitochondria from damage, further mitigating the risk of immune reactions triggered by the release of immunogenic substances from compromised mitochondria [248]. Hence, future research could leverage exosomes to reduce the risks associated with mitochondrial transplantation.

#### Concerns regarding the Long-term fate and safety of mitochondrial transplantation

The long-term survival, functional maintenance, and systemic safety of transplanted exogenous mitochondria constitute the core scientific questions determining their clinical translational value. While mitochondrial transfer is widely recognized for promoting wound healing through various mechanisms, the potential long-term biological effects following the introduction of foreign mtDNA into recipient cells require focused attention. The potential for interactions between exogenous mtDNA and the host nuclear genome to induce genomic alterations and subsequent adverse biological effects remains a subject of debate and differing interpretations [249–251], underscoring the importance of in-depth mechanistic investigations. Furthermore, the therapeutic efficacy of mitochondrial transplantation hinges critically on the efficiency of integration and functional reconstitution of the transplanted mitochondria within recipient cells. The specific molecular mechanisms governing the fusion and integration of exogenous mitochondria with the host’s endogenous mitochondrial network remain incompletely understood. Consequently, elucidating the critical conditions under which exogenous mitochondria exert therapeutic effects versus potential adverse outcomes represents a core issue that must be resolved before this technology can advance to clinical applications.

From the perspective of a therapeutic benefit, the core value of mitochondrial transplantation lies in its targeted intervention at the causal level. Upon entering target host cells, such as ECs and fibroblasts, healthy donor mitochondria can rapidly replace dysfunctional endogenous mitochondria, significantly increasing the efficiency of ATP synthesis in recipient cells and restoring their migration, proliferation, and secretory functions to thereby create favorable conditions for wound healing. Moreover, exogenous mitochondria can inhibit excessive ROS generation, downregulate proinflammatory signaling pathways such as NF-κB and TNF-α signaling, and promote macrophage polarization toward the M2 reparative phenotype, effectively disrupting the vicious cycle of “inflammation–oxidative stress”. Additionally, mitochondrial transplantation can improve EC function, upregulate the expression of proangiogenic factors, accelerate the formation of a mature vasculature and restore local blood perfusion, thereby facilitating wound re-epithelialization and the orderly repair of dermal tissue.

Based on these characteristics, the potential applications of mitochondrial transplantation in wound repair can be preliminarily inferred. First, mitochondrial transplantation is suitable for chronic inflammatory wounds lacking precancerous lesions or tumors. These wounds inherently suffer from mitochondrial dysfunction, insufficient proliferation, and impaired vascular maturation [15, 252, 253]. Appropriate dosing of transplanted mitochondria can specifically correct these pathological abnormalities, resulting in therapeutic activity at the causal level. Importantly, mitochondria do not harbor nuclear genomic mutations and lack the ability to undergo cellular transformation [254–256]. Furthermore, current animal studies and early clinical investigations have not indicated a direct tumorigenic risk from mitochondrial transplantation. Second, when standardized protocols with strict control over the mitochondrial dose and purity are used, acute adverse reactions such as local injection injury or transient oxidative bursts can be effectively mitigated, allowing the pro-healing advantages of these methods to be fully realized. Nevertheless, caution is warranted in specific scenarios. For patients who present with atypical hyperplasia, carcinoma *in situ*, or a history of malignancy and whose mitochondria carry no nuclear mutations and possess no direct oncogenic ability, vigilance is advised regarding the potential of the transplanted mitochondria to provide metabolic support to pre-existing abnormal cells. Additionally, patients with severe immunodeficiency or systemic inflammatory conditions require a careful assessment of the risk of an inflammatory response triggered by extracellular mitochondria. Moreover, the application of allogeneic mitochondrial transplantation in patients with autoimmune diseases demands even a stricter evaluation to avoid inducing immunogenic reactions that could exacerbate the underlying condition.

Crucially, the existing research on mitochondrial transplantation therapy has focused primarily on short-term efficacy. Systematic studies investigating the long-term fate of transferred mitochondria and chronic safety outcomes remain unreported, representing a significant bottleneck that impedes clinical translation. Recent short-term studies have confirmed that mitochondrial transplantation acutely improves the wound healing microenvironment with no obvious immediate risk. However, robust data are lacking concerning the long-term survival duration and functional stability of mitochondria within host tissues, as well as the potential for inducing chronic inflammation, abnormal hyperplasia, or excessive fibrosis with prolonged application. Therefore, future research must prioritize the following directions: (i) clarifying the long-term fate of transferred mitochondria in chronically inflamed host tissue, including their lifespan, patterns of functional decay, and clearance mechanisms; (ii) conducting long-term animal experiments and clinical follow-up studies to systematically evaluate the chronic safety of mitochondrial transplantation, with a particular focus on monitoring potential long-term risks such as tumorigenesis and pathological scarring; and (iii) delving deeper into the specific mechanisms by which extracellular mitochondria elicit inflammatory responses and identifying targeted regulatory strategies to mitigate potential risks and further optimize the risk–benefit ratio.

In summary, the therapeutic benefits of mitochondrial transplantation for repairing chronic nonhealing wounds are well supported and mechanistically defined. The balance of risks and benefits can be managed through strict definitions of application scenarios and standardized operational procedures. Under conditions excluding precancerous lesions and utilizing standardized autologous mitochondria, the core risk profile appears low. However, constrained by the limitations of existing short-term research, long-term safety remains uncertain. Future efforts must concentrate on the aforementioned research priorities to supplement the database with long-term studies, thereby providing a more robust theoretical and experimental foundation for the broader clinical adoption of this therapy.

#### Standardization and administration dosage in mitochondrial transplantation

Successful mitochondrial transplantation is influenced by multiple variables, including mitochondrial isolation methods, donor cell selection, the quantity of mitochondria transplanted, and the delivery modality [257]. The construction of a standardized system and the precise control of the administered dose constitute the core prerequisites for enabling its clinical translation and ensuring therapeutic safety and efficacy. While an exploratory framework for the entire “preparation–quality control–administration” process currently exists, a globally unified protocol is unavailable, and dosing lacks specific quantitative guidelines.

Standardization efforts focus on establishing uniform procedures for mitochondrial isolation, purification, storage, quality control, and delivery. During isolation, the tissue source and isolation method are critical considerations. MSCs, which are distributed across multiple adult tissues and are easily accessible, are considered promising donor cells. However, MSCs from different tissue origins exhibit variations in the respiratory status, mitochondrial transfer capability, and therapeutic efficacy [258]. A comparative study of the efficiency and quality of mitochondrial extraction from various mouse tissues revealed the lowest yields from muscle and peripheral blood, whereas mitochondria from liver and heart sources displayed more intact structure and higher purity, with liver-derived mitochondria showing a significantly lower ATP content and mitochondrial membrane potential than cardiac-derived mitochondria [259].

Following donor cell acquisition, critical parameters, including the processing time, homogenization buffer pH, centrifugation temperature, and centrifugal force gradients, must be optimized according to the tissue source to obtain sufficient quantities of high-quality mitochondria. Prolonged isolation processes can reduce mitochondrial viability and transplantation success rates [260], underscoring the importance of defining an appropriate and rapid isolation method.

During purification, traditional differential centrifugation results in relatively low recovery rates and purity. The integration of novel techniques, such as magnetic bead-based immune capture or microfluidic separation systems, is necessary to improve mitochondrial yield and purity and facilitate large-scale clinical application.

Mitochondrial preservation presents another significant challenge, as isolated mitochondria are fragile and lose respiratory function within approximately two hours after extraction [261]. Cryopreservation at −80°C impairs OMM integrity [262]. According to the available data, isolated mitochondria maintain viability and coupling for approximately one hour on ice, with subsequent storage significantly impacting engraftment efficiency [260]. Cryoprotectants, such as dimethyl sulfoxide (DMSO) [263] and trehalose [264], have been employed but may still lead to decreased functional, highlighting the need to develop novel preservation strategies that maintain mitochondrial stability and bioenergetic capacity.

Rigorous quality control is paramount, as the efficacy and safety of mitochondrial transplantation depend heavily on the structural integrity and functional activity of the donor mitochondria. The structural integrity of the outer and inner membranes and a stable mitochondrial membrane potential are prerequisites for mitochondrial survival and function. Only structurally intact mitochondria can be effectively internalized, integrated, and function within recipient cells [265]. Damaged mitochondria not only fail to restore respiration in recipient cells but also risk releasing DAMPs (mtDNA, ROS, and cardiolipin), triggering innate immune signaling and amplifying inflammatory responses [142]. Donor mitochondria must also possess the full OXPHOS capacity to effectively reverse aerobic respiration defects in recipient cells and restore energy metabolism [24]. Consequently, comprehensive quality control—assessing structural integrity, the mitochondrial membrane potential, and OXPHOS function—must precede transplantation to ensure efficacy and avoid potential harm to the patient.

Dose optimization requires a holistic approach that integrates the delivery route, characteristics of the target organ, disease type, and individual variations in patients, but a uniform standard has not yet been established. In wound healing, determining the “mitochondrial load” based on the wound area is key. Future research should focus on developing dose calculation models based on organ-specific physiological parameters, such as the tissue mass and local perfusion efficiency, to provide quantitative references for different target organ doses. In the future, when combined with large-sample and long-term clinical trials, AI-driven donor–recipient matching systems can be integrated to establish personalized dosage guidelines based on the “disease classification, target organ, and individual differences”.

### Future prospects

As an emerging therapeutic strategy, mitochondrial transplantation holds significant promise in multiple fields because of its unique capacity for the targeted repair of cellular energy metabolism and regulation of the tissue microenvironment. With the continuous advancement of related technologies, future developments will focus on clinical translation, gradually propelling this therapy from foundational research toward scaled clinical application and expanding its therapeutic scope and value.

First, the application of mitochondrial transplantation can extend from wound healing to the broader field of tissue regeneration. Existing evidence has demonstrated its considerable potential in repairing various tissue injuries, including brain injury [266], cardiac dysfunction [267], neural defects [268] and liver disease [269], and even in organ transplantation [270]. With further optimization of delivery systems and the elucidation of therapeutic mechanisms, the application of mitochondrial transplantation will be broadened to more tissue regeneration scenarios, and a multifaceted therapeutic system centered on “metabolic repair” that involves novel pathways for managing refractory tissue damage will be established.

Second, mitochondrial transplantation currently faces significant challenges in terms of specificity and efficiency. The data indicate that only ~10% of administered exogenous mitochondria reach target cells [261], with merely 1%–2% successfully internalized by these cells. Among those internalized, <10% evade degradation and successfully integrate into the cytoplasm [271]. Consequently, engineering strategies will be crucial for increasing therapeutic efficacy. Genetic editing and nanomodification techniques can improve mitochondrial activity, stability, and targetability. For instance, MDV nanogels carrying the Miro1 DNA can target macrophages and regulate mitochondrial transfer, promoting skeletal regeneration [272]. Encapsulating mitochondria in artificial scaffolds with magnetic nanoparticles enables targeted accumulation at cerebral hemorrhage sites upon intravenous administration and the application of an external magnetic field [273]. High-quality mitochondrial microfactories constructed by anchoring Prussian blue nanozymes enable the efficient delivery of therapeutic mitochondria [274]. Similarly, atomically engineered molybdenum disulfide (MoS_2_) nanoflowers can transform human MSCs into mitochondrial bioreactors, increasing their ability to undergo intercellular mitochondrial transfer [275]. Some researchers have also modified mitochondria with nanomotors and encapsulated them in enteric-coated capsules, achieving oral mitochondrial transplantation [276] and representing an innovative approach to delivery.

Third, future optimizations of delivery systems will focus on achieving controlled release and precise targeting at wound and target tissue sites, addressing the current limitations such as insufficient targetability, loss of activity, and nonspecific accumulation. Currently investigated delivery vehicles, including hydrogels, microneedles, and EVs, each offer distinct advantages: hydrogels enable slow release and prolonged local retention; microneedle patches circumvent the skin barrier for noninvasive, targeted delivery; and EVs leverage intrinsic targeting to reduce systemic clearance and increase the efficiency of mitochondrial delivery. Future research should integrate these approaches to develop multifunctional composite delivery systems. For example, combining hydrogels with microneedles achieves both “noninvasive delivery and controlled release” [230]. In addition, biomimetic modification with EV membranes, combined with ligand–receptor recognition, further improves the targeting accuracy [261]. This approach helps reduce the potential risks caused by off-target tissue accumulation and increases the safety and efficacy of therapy.

Finally, future investigations should further explore the mechanisms underlying the interaction between mitochondrial transfer and existing mainstream therapies, such as stem cell therapy and growth factor treatment, with a focus on constructing and optimizing synergistic therapeutic systems. Identifying synergistic effects and key molecular targets will enable the development of multidimensional therapeutic frameworks, such as combining “mitochondrial transplantation + stem cell therapy” or “mitochondrial transplantation + growth factor treatment”. These integrated approaches may represent more effective, comprehensive strategies for managing refractory tissue injuries.

## Conclusions

In this review, we began by outlining the effect of mitochondrial dysfunction on wound repair, systematically expounded on the physiological basis of mitochondrial transfer, discussed its triggers and regulatory factors, and focused on its potential role in wound healing. We then proposed translational pathways for mitochondrial transplantation in wound repair, providing insights into the clinical management of chronic, refractory wounds. Although mitochondrial transplantation faces numerous challenges, its promise in wound healing is well established, particularly when it is integrated with emerging technologies for potential breakthroughs. Crucially, clinical exploration of mitochondrial transplantation for wound repair currently remains limited, with an inadequate body of supporting research. Therefore, increased research investment is essential to advance this field.

## Abbreviation

mtDNA: mitochondrial DNA, ROS: reactive oxygen species, OXPHOS: oxidative phosphorylation, mGPDH: mitochondrial glycerol‑3‑phosphate dehydrogenase, ECM: extracellular matrix, F-actin: actin filaments, OMM: outer mitochondrial membrane, MV: microvesicle, Mfn-2: mitofusin-2, BAT: brown adipose tissue, MDV: mitochondrial-derived vesicle, GJ: gap junction, RFs: retraction fibers, PI3K: phosphatidylinositol 3-kinase, TNF-α: tumor necrosis factor-α, IL: interleukin, IFN-γ: interferon-γ, cADPR: cyclic adenosine diphosphate ribose, DAMP: damage-associated molecular pattern, RyR: ryanodine receptor, CD38: cluster of differentiation 38, CX43: connexin 43, hDF: human dermal fibroblast, HIF-1α: hypoxia-inducible factor-1α, MMP: matrix metalloproteinase, VEGFR2: vascular endothelial growth factor receptor 2, EC: endothelial cell, ATP: adenosine triphosphate, TNT: tunneling nanotube, Miro: mitochondrial Rho GTPase, BMSC: bone marrow mesenchymal stem cell, MSC: mesenchymal stem cell, EV: extracellular vesicle, IP3R: inositol trisphosphate receptor, MICOS: mitochondrial contact site and cristae organizing system, hMSC: human mesenchymal stem cell, UMSC‑apoVs: umbilical cord mesenchymal stem cell-derived apoptotic vesicle, IONP: iron oxide nanoparticle, pl-MT: platelet-derived mitochondria, HUVEC: human umbilical vein endothelial cell, OA: osteoarthritis, FAO: fatty acid oxidation, TCA: tricarboxylic acid, OCR: oxygen consumption rate, HK: hexokinase, PKM2: pyruvate kinase M2, CS: citrate synthase, IDH2: isocitrate dehydrogenase 2, ECAR: extracellular acidification rate, hFB: human fibroblast, VEGF: vascular endothelial growth factor, Ang-2: angiopoietin-2, SASP: senescence-associated secretory phenotype, CDK: cyclin-dependent kinase, β-GAL: β-galactosidase, TIMP: tissue inhibitors of metalloproteinase, TGF-β: transforming growth factor‑β, MRT: mitochondrial replacement therapy, Mito@euMVs: MSC-derived microvesicles containing functional mitochondria, LVEF: left ventricular ejection fraction, α-SMA: α-smooth muscle actin, IGF1R: insulin-like growth factor 1 receptor, MRPS5: mitochondrial ribosomal protein S5, h-UCMSC: human umbilical cord mesenchymal stem cells, ASC: adipose-derived stromal cell, iCMs: induced pluripotent stem cell-derived cardiomyocytes, ETC: electron transport chain, SMSC: skin mesenchymal stem cell, mPTP: mitochondrial permeability transition pore, MPMEC: mouse pulmonary microvascular endothelial cell, TRAK: trafficking kinesin protein, cAMP: cyclic adenosine monophosphate, PKA: protein kinase A, HSP60: heat shock protein 60, p4E-BP1: phosphorylated eukaryotic translation initiation factor 4E-binding protein 1, Col1A1: collagen, type1, alpha1, NAD+: nicotinamide adenine dinucleotide.
